# Motivational Barriers and Facilitators for Older Adults’ Engagement in Exercise during Depressive Episodes: A Network Meta-Analysis of Randomized Controlled Trials

**DOI:** 10.3390/healthcare12151498

**Published:** 2024-07-28

**Authors:** Mei-Ling Dai, Berne Ting, Ikbal Andrian Malau, Suet-Kei Wu, Chia-Ching Lin, Pan-Yen Lin, Min-Hsiung Chen

**Affiliations:** 1 Department of Nursing, Wei Gong Memorial Hospital, Miaoli 351498, Taiwan; 045064@tool.caaumed.org.tw; 2 Department of Nursing, Yuanpei University of Medical Technology, Hsinchu 30015, Taiwan; 3 Ph.D. Program for Aging, College of Medicine, China Medical University, Taichung 404328, Taiwan; u109048801@cmu.edu.tw; 4 Graduate Institute of Biomedical Sciences, College of Medicine, China Medical University, Taichung 404328, Taiwan; u110305142@cmu.edu.tw; 5 Mind-Body Interface Laboratory (MBI-Lab), China Medical University, Taichung 404328, Taiwan; 111059101@365.cmu.edu.tw; 6 Graduate Institute of Nutrition, China Medical University, Taichung 404328, Taiwan; 7 Department of Occupational Therapy, Wei Gong Memorial Hospital, Miaoli 351498, Taiwan; 045354@tool.caaumed.org.tw; 8 Department of Psychiatry, Wei Gong Memorial Hospital, Miaoli 351498, Taiwan; 9 Department of Nursing, Hsin-Sheng College of Medical Care and Management, Taoyuan 32544, Taiwan; 10 Department of Neurosurgery, Wei Gong Memorial Hospital, Miaoli 351498, Taiwan

**Keywords:** geriatric depression, exercise motivation, network meta-analysis

## Abstract

This study aims to assess the motivational factors influencing the participation of older adults in various exercise interventions during depressive episodes and to identify which types of exercise are most effective in alleviating depressive symptoms in this population. Therefore, randomized controlled trials (RCTs) focusing on exercise interventions and their impact on depression in older adult patients, identified by the terms “exercise” AND “depression” AND “elderly” OR “geriatric”, were selected from primary electronic databases to conduct this network meta-analysis (NMA). The primary outcome was the effect on depressive symptoms, while the secondary outcome was the comparison of dropout rates between the intervention groups and the usual care control groups, as a measure of sustained motivation and engagement. Standardized mean difference (SMD) values and the corresponding 95% confidence intervals (CIs) were computed for effect evaluation. This study protocol has been registered in IPLASY (INPLASY 202460035). The results of 31 RCTs with 3238 participants indicated that qigong (SMD −1.17, −2.28 to −0.06), Otago Exercise (SMD −1.15, −2.29 to −0.01), and yoga (SMD −0.88, −1.55 to −0.21) significantly alleviate depressive symptoms in older adults. Walking (SMD −0.82, −1.34 to −0.31) and strength training (SMD −0.67, −1.05 to −0.30) also showed significant effects. Aerobic, physical training, and tai chi had moderate effects, while multisport showed a weaker impact with no significant improvement. In summary, our research findings demonstrate that exercise can effectively alleviate depressive symptoms in older adults, with low dropout rates likely due to interconnected physiological, psychological, and social mechanisms. This is crucial for enhancing treatment strategies for older adults’ depression.

## 1. Introduction

Depression in older adults, particularly those aged 60 and above, is commonly referred to as geriatric depression. This condition warrants special attention not only due to its similarity to depression in the general adult population but also because of the unique physiological, psychological, and social challenges faced by this age group [1]. Geriatric depression often manifests as nonspecific physical discomfort, such as pain, malaise, and digestive issues, which can obscure the underlying emotional problems [2]. Additionally, cognitive decline induced by depression, such as memory loss and slowed thinking, may be mistaken for dementia, complicating accurate diagnosis and treatment [3,4]. Older adults encounter specific challenges that can exacerbate depressive symptoms, including widowhood, retirement, and shrinking social circles [5]. Furthermore, episodes of depression in older adults are often compounded by a lack of motivation for physical activity, which can further deteriorate their mental and physical health [6]. Recognizing the challenges faced by this demographic, it is crucial to consider the role of exercise interventions. Exercise can serve as a powerful tool to counter the effects of depression by improving physiological health and enhancing psychological well-being. Engagement in regular physical activities has been shown to mitigate the symptoms of depression and improve overall quality of life in older adults [7].

Multiple exercise interventions are highly suitable for older adults, addressing their physiological, psychological, and social needs [8]. Tai chi, a gentle exercise combining deep breathing and flowing movements, enhances muscle strength and flexibility and reduces the risk of falls, and its meditative nature helps alleviate stress. Participating in community tai chi classes also aids in building social networks, reducing feelings of loneliness [9,10]. Yoga, which integrates postures, breath control, and meditation, improves physical flexibility and strength, promotes mental tranquility and emotional stability, and has shown positive effects on neural adaptability [11]. The Otago Exercise Program, designed specifically to improve balance and prevent falls in older adults, includes strength and balance training that can be performed at home, effectively enhancing self-efficacy and physical function [12]. Walking, a low-intensity aerobic exercise suitable for older adults of varying fitness levels, benefits cardiovascular health and sleep quality, and walking with others enhances social interaction [13,14]. Qigong involves breathing, movement, and meditation techniques, boosting physical fitness while helping with psychological relaxation and stress reduction [15,16]. Jogging and aerobic exercises improve cardiovascular function and muscle endurance, and rapidly increase serotonin levels in the brain, improving mood [17]. Strength training enhances muscle strength and bone health, increasing the self-efficacy of older adults [18].

Given the multifaceted benefits of various exercise interventions in addressing the physiological, psychological, and social needs of older adults, it is essential to systematically compare and evaluate these interventions to identify the most effective strategies. To achieve this, network meta-analysis (NMA) is a statistical method aimed at simultaneously evaluating the effects of multiple interventions to identify the most effective treatment option [19,20]. Unlike traditional meta-analysis, NMA enables the ranking of interventions, allowing for the assessment of their relative efficacy. As one of the highest levels of evidence, NMA plays a crucial role in formulating clinical strategies and practice guidelines. This technique constructs a network model by aggregating interventions from different studies, and this technique enables the comparison and ranking of these interventions based on their effectiveness. Direct comparisons occur in studies that explicitly contrast various interventions, while in the absence of direct comparisons, indirect comparisons are made using common comparators. NMA rigorously examines statistical differences in direct and indirect evidence to ensure the coherence and reliability of the analysis [21,22]. The aim is to assess the motivation of older adults to participate in various interventions during episodes of depression and to identify the most effective exercises for alleviating depressive symptoms. By evaluating and ranking the impact of these exercise interventions, we hope to determine the optimal strategies for improving treatment outcomes for older adults with depression.

## 2. Materials and Methods

In adherence to the guidelines set by the Preferred Reporting Items for Systematic Reviews and Meta-Analyses, particularly for network meta-analyses (PRISMA NMA) [23], this study was carefully designed. The study protocol has been officially registered under the registration number INPLASY202460035 with the International Platform of Registered Systematic Review and Meta-Analysis Protocols (INPLASY).

### 2.1. Database Search and Identification

We conducted an extensive search across four electronic databases—PubMed, Web of Science, Embase, and Cochrane Library—to identify relevant studies. Covering literature up to January 2024, we employed Boolean operators to search for the terms “exercise” AND “elderly depression” AND “randomized controlled trials.” We aimed to review and synthesize studies on the effects of exercise interventions on older adults’ depression. The first phase, we screened duplicates and excluded irrelevant studies. Following this, we conducted a manual search by checking the reference lists of several review articles for additional relevant studies. Two authors (Dai and Ting) independently reviewed the titles and abstracts of the screened articles for relevance. In cases of disagreement, a third author (Lin) intervened to reach a consensus and complete the selection process. These systematic methods ensured that each included study met the defined eligibility criteria.

### 2.2. Inclusion and Exclusion Criteria

This NMA adheres to the PICO model as the population, intervention, comparison, and outcome guidelines: P—older adult patients with depression; I—exercise therapy; C—any control group or alternative non-pharmacological interventions; O—standardized assessments of depression score in the older adults. Articles included in the analysis had to meet the following criteria: (1) randomized controlled trials (RCTs); (2) the intervention group received exercise therapy, which included commonly practiced exercises for older adults such as yoga, tai chi, and qigong, and control group received standard care, no treatment, usual low-intensity activity, or non-interventional exercise; (3) outcome measures included depression assessment indices; and (4) participants were aged 60 years or older. Articles were excluded based on the following criteria: (1) medical protocols, review articles, case reports, conference papers, medical letters, medical reviews, pilot studies, and preliminary results of trials; (2) studies where exercise therapy was combined with other therapies (e.g., exercise with cognitive therapy) or as part of complementary therapy; (3) control groups that included any form of exercise; and (4) studies without outcome data for analysis. Utilizing the full texts of eligible articles, the final network meta-analysis was conducted.

### 2.3. Model Construction for Network Meta-Analysis

In constructing the model for this NMA, we adhered to specific criteria. To minimize excessive heterogeneity, pairwise comparisons focused on exercise versus exercise or exercise versus standard care. Comparisons involving exercise combined with various invasive treatments (e.g., electrotherapy, laser light injections) or complementary and alternative therapies were excluded. Including these additional treatments could have led to diverse network geometries, resulting in potentially inconsistent analysis outcomes due to the variety of interventions considered [24]. In our study, the classification of exercise types was based on discussions about the actual exercise prescription content between two authors (Dai and Ting). Any differences in classification opinions were resolved through discussion with a third author (Lin) to reach a consensus.

### 2.4. Risk of Bias Assessment

The methodological quality of the included studies was evaluated using the Cochrane Collaboration’s risk of bias tool for randomized trials (RoB 2, version 2, London, UK) [25]. This tool evaluated key aspects of study quality, including the randomization process, adherence to intervention protocols, handling of missing outcome data, accuracy of outcome measurement, potential for selective reporting, and overall risk of bias.

### 2.5. Primary Outcome: Improvement of Depression in the Older Adults

The primary outcome of the exercise intervention was the improvement of depressive symptoms in older adult patients, assessed through the standardized mean difference (SMD). The Geriatric Depression Scale (GDS) was the preferred measure due to its demonstrated significance in evaluating depressive symptoms [26]. Secondary options included other scales such as the Beck Depression Inventory (BDI) [27], and the Hamilton Depression Rating Scale (HAMD) [28]. This structured approach to selecting assessment scales aimed to achieve consistency and precision in evaluating depressive symptoms across the study population.

### 2.6. Secondary Outcome: Differences in Dropout Rates

The secondary objective of this study was to evaluate the differences in dropout rates between participants undergoing exercise interventions and those in the control group as a measure of sustained motivation and engagement. The “risk difference (RD)” quantified the absolute difference in the proportion of participants who withdrew from the study in each group. Understanding risk differences was crucial for determining which exercises were most effective at keeping older adult participants engaged, thereby enhancing the overall success of the intervention in treating depressive symptoms [29].

### 2.7. Data Extraction, Handling, and Transformation

The data extraction process included collecting participants’ demographic data, study design details, specific conditions of the exercise interventions, and study outcomes. In instances where essential data was absent from published studies, we strove to acquire this information directly from the study authors. We adhered to data management protocols specified in the Cochrane Handbook and drew guidance from established medical research literature [21,30,31,32]. To ensure consistency and accuracy, any discrepancies between authors during data extraction were resolved through discussion and consensus, and, if necessary, by consulting a third author. This approach ensures uniform and meticulous handling of data, contributing to the reliability and validity of our NMA findings. 

### 2.8. Statistical Analysis

To account for the variability in types of exercise interventions, we utilized a random effects model [33]. Our analysis employed frequentist methods using MetaInsight (version 5.2.1; Complex Reviews Support Unit funded by the National Institute for Health Research (NIHR), London, UK). Statistical analysis was performed with the netmeta package, integrated into the online NMA platform in R [34]. Initially, forest plots and network diagrams were generated to illustrate pairwise comparisons within the studies. Subsequently, forest plots were created to summarize the standardized mean differences in depression improvement and the risk differences in dropout rates among older adult patients with depression. These plots compared the impact of each type of exercise intervention against the control group [35]. The results were expressed as point estimates and 95% confidence intervals [35]. We ranked the exercise therapies based on their effectiveness, presenting the results of direct and indirect comparisons in tables. Specific statistical tests assessed data inconsistency, with bilateral *p*-values less than 0.05 indicating statistical significance.

### 2.9. Sensitivity Analysis Methods

We performed two separate sensitivity analyses to ensure the reliability of our findings. The first analysis involved systematically excluding each study to assess whether any individual study had a significant influence on the overall outcomes. This method entailed stepwise elimination of each study, followed by an evaluation of how these removals affected the conclusions and the comparative effects of the interventions. The second sensitivity analysis examined the correlation coefficient used in the pre-and post-assessments of depression in older adults. Initially, we applied a correlation coefficient of 0.8, as recommended in the Cochrane Handbook for its conservative estimation of correlation in longitudinal data, which minimizes the risk of overestimating treatment effects [36]. To further validate our findings, we performed additional sensitivity analyses using different correlation coefficients, particularly ranging from 0.5 to 0.8 [37]. These ranges were chosen based on existing literature suggesting that lower coefficients might reflect more realistic scenarios of less consistent changes over time, thus providing a more stringent test of the interventions’ effectiveness. In this process, we recalculated the effect size for changes in depression in older adults using a lower correlation coefficient of 0.5 [37] to assess how variations in the correlation coefficient influenced the direction and magnitude of the results, statistical significance, and the comparative effects of the interventions.

### 2.10. Publication Bias

We assessed potential publication bias following the guidelines outlined in the Cochrane Handbook for Systematic Reviews of Interventions [21]. We generated a funnel plot for comparisons involving control groups using Comprehensive Meta-Analysis software, version 4 (Biostat, version 4, Englewood, NJ, USA). Additionally, to identify significant publication bias, we employed the Egger regression test. 

## 3. Results

### 3.1. Study Identification and Network Model Construction

**Figure 1:**
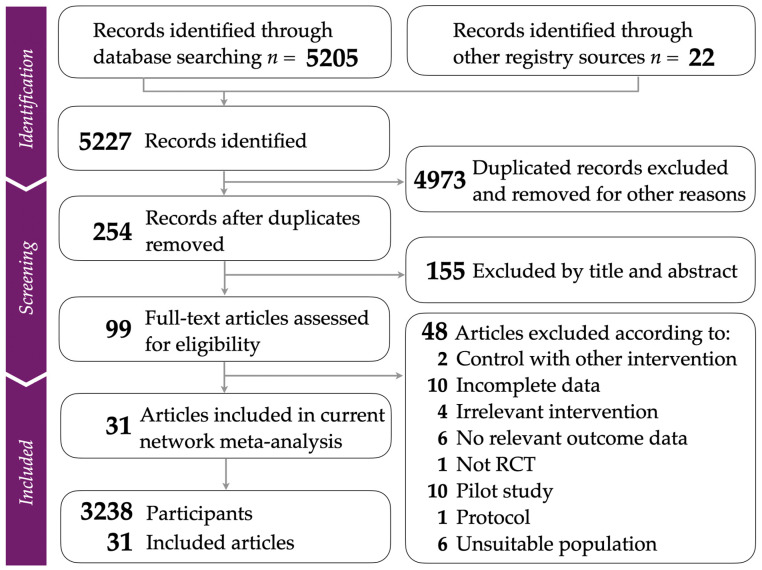
Flowchart illustrating the study selection process in compliance with PRISMA guidelines.

Our study rigorously followed the PRISMA guidelines, as depicted in the flow diagram in Figure 1. For additional details, the PRISMA NMA checklist is available in Supplementary Table S1. The total number of articles retrieved from various databases was specified in Supplementary Table S2. After removing duplicates and excluding irrelevant studies based on titles and abstracts, we included 31 randomized controlled trials (RCTs) [38,39,40,41,42,43,44,45,46,47,48,49,50,51,52,53,54,55,56,57,58,59,60,61,62,63,64,65,66,67,68]. Supplementary Table S3 offers comprehensive details on the articles excluded during the final selection phase, along with the reasons for their exclusion. 

**Figure 2:**
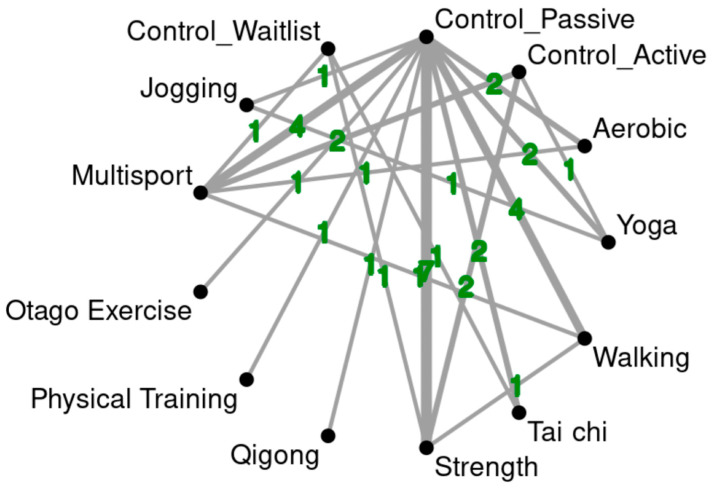
This network diagram depicts the post-intervention effects of various exercise interventions on alleviating depression in older adults. The size of the nodes and the thickness of the lines represent the number of trials included in our study.

A total of 31 RCTs were included, involving 3238 participants. The interventions identified in these studies were categorized into the following groups: aquatic exercise, jogging, multisport, Otago Exercise, physical training, qigong, strength, tai chi, walking, walking + aerobic exercise, and yoga. The network model representing these various intervention methods is shown in Figure 2. The general characteristics of the included studies provide a broad summary, including the authors, publication years, and countries of origin. Detailed descriptions of the study designs are provided to ensure a clear understanding of the methods used. Emphasis was placed on both the experimental and control groups, recording key details of all studies, such as the number of participants, dropout rates, average age, and specifics of the interventions (e.g., type of exercise). Information about the control groups, including the nature and description of the control strategies, was also included. Additionally, the study specifies the minimum Metabolic Equivalent of Task (MET) value estimate, duration, frequency, and schedule of the interventions, as well as the total treatment time. The outcomes evaluated in each study are presented in Table 1.

### 3.2. Studies’ Quality and Risk of Bias Assessment

Analysis of the methodological quality across the 31 studies revealed the following details: In the randomization process, all studies showed low risk of bias, with 100% (31/31) maintaining stringent randomization standards. Intervention adherence varied, with 77% (24/31) demonstrating low risk and 23% (7/31) showing some risk, indicating a need for improved adherence protocols in a portion of the studies. Missing outcome data was impeccably managed in all studies, as evidenced by 100% (31/31) scoring low-risk, which enhances the credibility of the study results. Outcome measurement was generally reliable with 65% (20/31) at low risk and 35% (11/31) at some risk, suggesting some studies could benefit from more meticulous measurement strategies. Selective reporting was excellently handled, with all studies transparently reporting outcomes, resulting in 100% (31/31) at low risk. The overall risk of bias was moderately concerning, with 65% (20/31) categorized as low-risk and 35% (11/31) as some-risk (detailed in Figure S1). Despite the generally low risk in most categories, the varying results in intervention adherence and overall risk of bias highlight areas where certain studies could enhance their methodological rigor. Detailed risk evaluations for each category can be referred to in Table S4.

### 3.3. Main Outcome: Effective Exercise Interventions for Alleviating Depression in Older Adults

**Figure 3:**
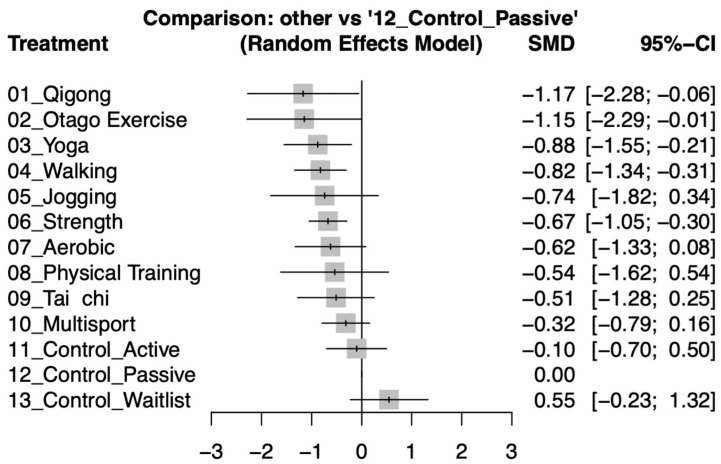
Forest plots depicting the standardized mean differences (SMD) in depression symptom improvement among older adults, comparing various exercise interventions to control groups after the intervention period.

This forest plot illustrates the effectiveness of various exercise interventions in alleviating depressive symptoms in older adults, using the SMD and its 95% CI. Qigong demonstrates the strongest improvement in depressive symptoms (SMD −1.17, CI −2.28 to −0.06), followed closely by Otago Exercise (SMD −1.15, CI −2.29 to −0.01) and yoga (SMD −0.88, CI −1.55 to −0.21). Walking and jogging also show significant effects (SMD −0.82, CI −1.34 to −0.31 and SMD −0.74, CI −1.82 to 0.34, respectively). Strength training and aerobic exercises are effective as well, with SMD −0.67, CI −1.05 to −0.30 and SMD −0.62, CI −1.33 to 0.08. Physical training and tai chi have moderate effects but with wide CIs (SMD −0.54, CI −1.62 to 0.54 and SMD −0.51, CI −1.28 to 0.25). Multisport has a weaker effect (SMD −0.32, CI −0.79 to 0.16). Control Active and Control Waitlist show no significant improvement (SMD −0.10, CI −0.70 to 0.50) and an increase in symptoms (SMD 0.55, CI −0.23 to 1.32), respectively. The results suggest that traditional low-intensity exercises like qigong, Otago, and yoga are most effective in improving depressive symptoms among older adults, potentially due to their dual benefits on physical health and mental stress relief (Figure 3). Detailed pairwise comparisons between study arms, as outlined in the individual studies, are shown in Figure S2.

Table 2 displays the results from the pairwise meta-analyses above the diagonal line and the network meta-analysis (NMA) results below it. The effect sizes, represented by standardized mean differences (SMD), are accompanied by 95% confidence intervals (CIs).

### 3.4. Secondary Outcome: Differences in Dropout Rates

**Figure 4:**
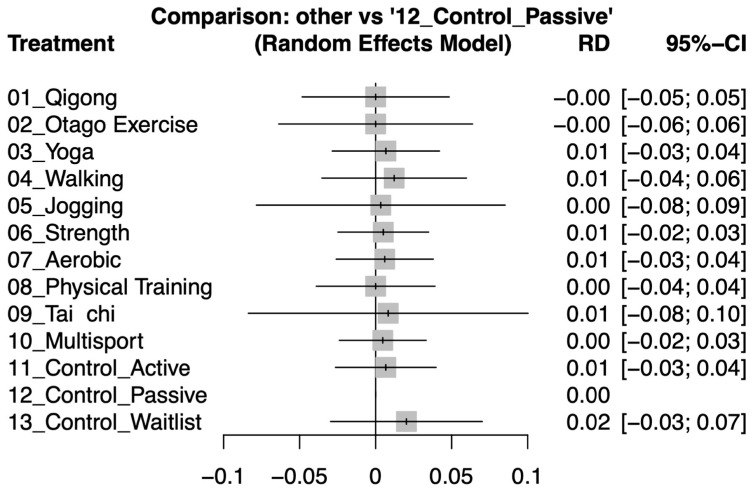
Forest plots demonstrating the risk differences (RD) in dropout rates among older adults, comparing various exercise interventions with control groups post-intervention.

The analysis indicated no significant differences in dropout rates among the various exercise interventions compared to the control groups, as evidenced by the risk differences (RD) with all confidence intervals crossing zero (refer to Figure 4). This lack of significant variation suggests that motivation and commitment to participate did not differ substantially across different types of exercises and control activities. Such findings imply that the exercises are equally manageable and do not adversely affect participant retention. For a more granular analysis of direct comparisons between specific groups within the studies, refer to Figure S3.

### 3.5. Inconsistency Testing

In the studies examining the effects of exercise interventions on depressive symptoms in older adults, we constructed a network by establishing nodes and performing both direct and indirect comparisons to evaluate consistency. Table S5 contains the results of inconsistency tests for the impact of various exercise interventions on improving depressive symptoms in older adults. Dropout rate information is provided in Table S6. The *p*-values reported in both tables are greater than 0.05, indicating no significant inconsistency between the comparisons.

### 3.6. Sensitivity Analysis

In the sensitivity analysis excluding individual studies, the data highlighted the significant effects of exercise interventions on depression in older adults. We initially selected a correlation coefficient of 0.8 based on prior studies and expert consensus in the field, reflecting a strong assumed correlation between pre-and post-intervention measurements. With a correlation coefficient of 0.8, qigong, Otago Exercise, yoga, and walking all demonstrated significant reductions or improvements in depressive symptoms (Figure 3). In another sensitivity assessment, we updated the network comparisons by changing the pre- and post-correlation coefficient from 0.8 to 0.5. This adjustment showed a weakening of effect sizes and a widening of the 95% confidence intervals, indicating a decrease in the precision of the results. Specifically, qigong and Otago Exercise were no longer significant after adjusting the correlation coefficient to 0.5. However, the direction and ranking of most exercise interventions remained largely consistent (Figure S5). These changes underscore the importance of choosing correlation coefficients in sensitivity analysis and highlight the necessity of considering multiple correlation coefficients when conducting statistical analyses to comprehensively evaluate the impact of exercise interventions on depression in older adults. These combined analyses emphasize the reliability of our study results, demonstrating their stability under conditions of selective study inclusion or exclusion and variations in analytical assumptions. For more detailed insights, refer to Figure S4(1–31).

### 3.7. Publication Bias

Egger’s test applied to the analysis of the funnel plot yielded a *p*-value of 0.001, indicating significant publication bias (Figure S6).

## 4. Discussion

### 4.1. Main Findings and Clinical Significance

Our NMA highlights that low-intensity exercises such as qigong, Otago Exercise, and yoga are the most effective at alleviating depressive symptoms in older adults, suggesting that these activities are particularly well-suited to their capabilities and interests. Walking and jogging also provide significant benefits, while strength training and aerobic exercises show moderate improvements. Physical training, tai chi, and multisport, although less effective, still offer positive outcomes. Importantly, our analysis revealed no significant differences in dropout rates across various exercise interventions and control groups. This finding suggests that the motivational aspects of these exercises are robust, as participants are likely to continue with the interventions irrespective of the type. Such high retention rates indicate that these exercises are not only effective but also appealing and manageable for older adults. These insights should guide caregivers and healthcare providers in making informed decisions about incorporating specific exercises into therapeutic regimens, optimizing both engagement and therapeutic outcomes. 

### 4.2. Significance of Results in the Context of Current Research

Prior to our study, the latest comprehensive network meta-analysis was published by Tang et al. in “BMC Geriatrics”. This analysis synthesized 47 studies involving 2895 participants. Their intervention analysis of seven types of exercise found that walking was the most effective in alleviating depressive symptoms in older adults, with a primary focus on comparing exercise dosages. However, there were discrepancies between their ranking results and ours [69]. Additionally, Correia et al. published a meta-analysis in “Sports Health” analyzing the effects of different exercise regimens on adult depression. Their study showed significant effects of moderate-intensity exercise and interventions exceeding 150 min per week [70]. Unlike their findings, our study emphasizes low-intensity exercises as more suitable for older adults, possibly due to differences in the age and baseline fitness levels of the populations studied. Our sample exclusively comprised older adults, who may respond differently to exercise regimes than the mixed-age adult population in the Correia study. Furthermore, Mahmoudi A et al. published a meta-analysis in “Biological Research for Nursing”, which analyzed aerobic training, resistance training, or combined training, incorporating 18 studies with 1354 participants. Their results showed that exercise significantly reduced depressive symptoms, but there was a lack of information on other types of exercise [71]. Our comprehensive analysis includes a wider array of exercise types, providing a more holistic view of the potential benefits across different physical activities.

In our study, we concluded that qigong and the Otago Exercise Program (OEP) are the most effective exercise types for treating depression in older adults, followed by yoga, walking, and strength training. This study explores the effects, comparisons, and rankings of different exercise types in older adults’ depression research. Our research directly compares and ranks the impact of various exercise interventions on older adults’ depression, treating each exercise intervention as a benchmark for the study. However, some studies are based on self-reported surveys and lack prospective designs with clear definitions of the types of exercise interventions used (Table S3). Moreover, while some systematic reviews include older adult patients with depression before and after interventions, they also encompass other comorbid conditions [72,73]. Therefore, exercise therapy offers potential benefits in improving depressive symptoms among older adults. We seek to understand not just the therapeutic effects but also the motivational drivers behind sustained participation in these exercise programs. By exploring how different exercises cater to the preferences and physical capabilities of older adults, this research aims to fill existing gaps in the literature and provide a more comprehensive understanding of how to design exercise interventions that optimize both engagement and clinical outcomes for older adult depression patients.

### 4.3. Possible Explanations for the Observed Results

The observed effects of exercise on improving older adult depression patients can be elucidated through a series of interconnected physiological, psychological, and social mechanisms. Collectively, these mechanisms form a comprehensive intervention strategy aimed at fundamentally ameliorating depressive symptoms and enhancing the quality of life for patients. Furthermore, these mechanisms also serve to increase the motivation of older adult patients with depression to engage in physical activity.

Each type of exercise has its unique benefits suitable for older adults, allowing them to choose based on their individual health conditions and preferences [74]. Aquatic exercises are joint-friendly, effectively reducing joint stress, and the buoyancy and resistance of water provide safe strength training [75], and the exercise helps improve cardiovascular function and muscle strength and has a positive impact [76]. Tai chi, known for its gentle movements, significantly enhances balance and flexibility in older adults. It also plays a critical role in reducing stress and promoting psychological well-being [77]. The Otago Exercise Program, designed to prevent falls in older adults, includes balance and strength training that can be performed at home, effectively enhancing self-efficacy and physical function [12]. Walking, the simplest form of aerobic exercise, improves cardiovascular health and increases brain blood flow, promoting mental health [72]. Yoga, through various postures and breath control, enhances flexibility and muscle strength while promoting mental relaxation, proving effective against depression [78,79]. Moderate strength training can increase muscle mass and strength, improve posture, and help boost basal metabolic rates, crucial for maintaining both physical and mental health [18]. These exercises all have the potential to improve mental health, especially for older adults, who should choose the type of exercise that suits them best. These exercises support physiological improvement to varying degrees, helping to combat depression.

Regular exercise effectively combats depression in older adults by enhancing self-esteem, improving mood, and increasing social engagement. It not only boosts cognitive functions and sleep quality but also strengthens the overall sense of well-being as older adults experience improvements in their physical health and social life [80,81]. Exercise also promotes the release of endorphins in the brain, a natural mood enhancer that helps alleviate stress and depressive feelings [82]. Physical activity helps improve sleep quality, which directly affects cognitive function and psychological state [83]. Additionally, regular exercise has been shown to improve memory and executive function in older adults [56]. Suitable forms of exercise include aerobic activities such as walking, jogging, cycling, and aquatic aerobics, which enhance cardiovascular function while releasing endorphins to improve mood and reduce stress [84]. Strength training with dumbbells, resistance bands, or bodyweight exercises helps build muscle and improve physique, significantly enhancing self-esteem and self-efficacy [80]. Participating in group activities like tai chi, yoga, or dance classes not only provides physical exercise but also offers opportunities for social interaction, helping to build social support networks, which is very effective in improving mood and reducing feelings of depression [85]. Through these forms of exercise, older adults can gain psychological benefits in multiple ways, effectively combating depressive symptoms. Regular physical activity improves not only physical health but also psychological and social well-being, which is crucial for enhancing overall quality of life.

When considering the social mechanisms of exercise’s impact on older adults’ depression, the difference between group activities and individual activities is an important factor [86]. Group activities provide a natural social environment where participants can connect with others and share experiences, which helps reduce loneliness and social isolation in older adults [87]. Through group exercise, participants can feel collective support and encouragement, and this support network has a significant positive impact on mental health, especially in combating depression. Moreover, group settings often stimulate greater participation enthusiasm, and sustained motivation, as group members encourage each other to achieve exercise goals together. Individual activities allow for greater flexibility and personalization, letting participants choose the most suitable type and intensity of exercise according to their health conditions and interests [88]. For some older adults’ ideals, solitary exercise offers an opportunity for contemplation and self-reflection, beneficial for psychological recovery [89]. Individual activities also allow for more flexible scheduling and integration into daily life. When designing exercise programs for older adults with depression, combining group and individual activities based on individual needs and preferences can be advantageous [74]. For instance, a program might include group tai chi or aquatic exercise classes several times a week, combined with individual walking or yoga practice at home. Such a combination maximizes the benefits of social interaction while retaining the flexibility and personalization of individual activities. In this way, exercise becomes not just a physical therapy but a comprehensive social and psychological treatment tool, improving the overall quality of life and mental state of older adults.

In summary, the improvements in depression brought about by exercise are multifaceted, encompassing physiological regulation, psychological empowerment, and social support, forming a comprehensive treatment strategy. The synergistic effects of these mechanisms explain why exercise can be an effective tool against depression. Additionally, these mechanisms enhance the motivation of older adults with depression to participate in physical activity, providing strong theoretical support for the positive outcomes observed in this study.

### 4.4. Study Limitations

Our NMA indicates that exercise therapy has potential benefits for improving depressive symptoms in older adults. However, several limitations should be considered when interpreting these results. Firstly, the heterogeneity in depressive characteristics due to subjects from different backgrounds and age groups adds complexity to the analysis. Additionally, the small sample sizes in some studies may introduce bias. A significant concern is the higher dropout rate among older adults, which could impact the accuracy of the results. To confirm the reliability of our findings, we reviewed the 31 included studies and validated that no particular study or study group disproportionately influenced the overall results through consistency checks and sensitivity analyses. Despite these challenges, our findings have important implications for the daily care and mental health of older adults with depression. 

## 5. Conclusions

In summary, despite various discussions on the etiology and pathological mechanisms of depression in older adults, our research findings clearly indicate that low-intensity exercises such as qigong, the Otago Exercise Program, and yoga significantly alleviate depressive symptoms in older adults. Additionally, activities like walking and jogging have shown substantial positive effects. Moreover, exercise intervention can improve older adults’ depression without increasing the risk of dropout, highlighting the critical role of exercise motivation. This is essential for improving treatment strategies for older adults’ depression.

## Figures and Tables

**Table 1:** Summary of the effectiveness of exercise interventions in improving depression in older adults, including details from the conducted trials.

			Sample				Intervention Group			Control Group			
Authors & Year	Country	Comparison	*n*	Dro- pouts	Age, Mean (SD)	Gender ♀ (%)	IX Type	Dosage	Min. MET/ Week	Ctl. Type	Descriptions	Duration	Outcomes
Aguinaga et al., 2018 [38]	USA	Strength Control	158 149	34 26	70.62 (5.02) 71.43 (5.25)	71% 82%	Strength	Freq. of light, mod., & vig. exercise ≥ 15 min/sess., weekly in leisure time.	-	Passive	Educational	24 w	HADS
Belvederi Murri et al., 2015 [39]	Italy	Aerobic Multisport Control	42 37 42	0 0 0	75.00 (6.20) 75.00 (6.30) 75.60 (5.60)	69% 68% 76%	Aerobic Multisport	60 min/sess., 3 x/w	2160	Active	Pharmacological treatment only	24 w	HADS
Boström et al., 2016 [40]	Sweden	HIFE Control	93 93	10 12	84.40 (6.20) 85.90 (7.80)	76%	Strength	45 min/sess., total 40 sess.	630	Passive	Activities of daily living	16 w	GDS MADRS
Brittle et al., 2009 [41]	UK	Exercise Control	20 18	3 5	87.00 (6.99) 82.00 (9.98)	79% 64%	Multisport	40–60 min/sess., 2 x/w	480	Passive	Usual care	12 w	SADQ
Chang et al., 2018 [42]	South Korea	Aerobic Control	47 46	2 3	77.80 (6.60) 77.80 (6.60)	86% 88%	Multisport	30 min/sess., 3 x/w	540	Passive	Social	12 w	GDS
Chen et al., 2009 [43]	Taiwan	Yoga Control	67 72	3 6	69.20 (6.23)	73%	Yoga	70 min/sess., 3 x/w	525	Passive	Usual care	24 w	TDQ
Chen et al., 2015 [44]	Taiwan	Walking exercise Control	58 58	8 7	64.76 (11.28) 63.57 (10.54)	55% 51%	Walking	40 min/sess., 3 sess./week, and weekly exercise counseling	300	Passive	Usual care	12 w	HADS
Chen et al., 2021 [45]	China	OEP Control	31 31	1 2	84.59 (4.21) 84.75 (5.41)	83% 60%	OEP	30 min/sess., 3 sess./w	315	Passive	Activities of daily living	12 w	GDS-15
Cheng et al., 2012 [48]	China	Tai chi Control	12 12	0 0	81.00 (7.70) 82.50 (7.10)	50% 75%	Tai chi	60 min/sess., 3 x/w	540	Passive	Social	24 w	GDS
Chou et al., 2004 [46]	China	Tai chi Waitlist	14	0	72.60 (4.20)	50%	Tai chi	45 min/sess., 3 x/w	405	Waitlist	Waitlist	12 w	CES-D
Conradsson et al., 2010 [47]	Sweden	Exercise Control	91 100	2 1	85.3 (6.1) 84.2 (6.8)	74% 72%	Strength	45 min/sess., 5 sess./two weeks (total 29 sess.)	1142	Passive	Activities of daily living	12 w	GDS-15
Danhauer et al., 2022 [49]	USA	Yoga Control—CBT	110 100	0 0	66.30 (4.90) 66.70 (5.70)	82% 90%	Yoga	75 min/session, 1 x/2 w	94	Active	CBT	11 w	PROMIS-29 Depression
de Lima et al., 2019 [50]	Brazil	Resistance training Control	17 16	0 0	66.2 (5.5) 67.2 (5.2)	-	Strength	30–40 min/sess., 2 sess./w	210	Active	Pharmacological treatment only	20 w	HRDS
Deus et al., 2021 [51]	Brazil	Strength Control	101 110	20 34	67.27 (3.24) 66.33 (3.88)	43% 47%	Strength	3 x/w	-	Waitlist	Waitlist	24 w	BDI
Gary et al., 2010 [52]	USA	Home-based exercise Control	20 17	3 3	65.80 (13.50)	57%	Walking	60 min/sess., 3 x/w	450	Active	CBT Usual care	12 w	HAMD
Huang et al., 2015 [53]	Taiwan	Strength Control	19 20	0 0	76.53 (5.94)	53%	Strength	50 min/sess., 3 x/w	525	Active Passive	CBT Usual care	12 w	GDS
Lavretsky et al., 2022 [54]	USA	Tai chi Control	89 89	27 26	69.20 (6.90) 69.40 (6.20)	70% 75%	Tai chi	60 min/sess., 1 x/w	180	Passive	Educational	12 w	GDS
Lok et al., 2017 [55]	Turkey	Mixed aerobic exercises Control	80	0	65–90	45%	Multisport	70 min/sess., 4 x/w	1680	Waitlist	Waitlist	10 w	BDI
Makizako et al., 2020 [56]	Japan	Strength Control	27 28	3 1	73.10 (5.50)	51%	Strength	90 min/sess., 1 x/w	315	Passive	Educational	24 w	GDS
McMurdo et al., 2001 [57]	UK	Aerobic + Strength Control	87	0	65.30 (4.30)	59%	Aerobic + Strength	45 min/sess., 3 x/w	1283	Passive	Educational	10 w	GDS
Moraes et al., 2020 [58]	Brazil	Aerobic Strength Control	9 9 7	8 8 5	70.88 (5.940 72.89 (7.06) 69.28 (5.28)	-	Aerobic + Strength	5 min warm-up, 20 min exercise; 3 sets of 8–12 reps for major muscle groups	765	Passive	Activities of Daily Living	12 w	HAMD BDI
Ng et al., 2017 [59]	Singapore	Physical Training Control	48 50	0 0	70.3 (5.25) 70.1 (5.02)	-	Physical Training	90 min, 2 days/w	900	Passive	Usual care	12 m	GDS
Seino et al., 2017 [60]	Japan	IIG DIG	38 39	0 0	74.9 ± 5.3 74.3 ± 5.6	37% 26%	Strength	100 min/sess., 2 x/w	700	Passive	DIG	12 w	GDS
Shahidi et al., 2011 [61]	Iran	Jogging Laughter Yoga Control	60	0	65.70 (4.20) 65.50 (4.80) 68.40 (6.30)	100%	Jogging yoga	30 min/sess., 10	2100 750	Waitlist	Waitlist	10 times	GDS
Shahtahmassebi et al., 2022 [62]	Iran	Trunk Strengthening Walking	32 32	0 0	70.1 (7.7) 69.4 (7.3)	57% 63%	Strength	3 sess./w	-	Passive	Activities of daily living	18 w	GDS
Singh et al., 1997 [63]	USA	Strength Control	32	0	70.00 (1.50) 72.00 (2.00)	71% 53%	Strength	45 min/sess., 3 x/w	472.5	Passive	Educational	10 w	GDS
Singh et al., 2005 [64]	Australia	Strength—high-intensity Strength—low-intensity Control	18 17 19	2 3 1	69.00 (5.00) 70.00 (7.00) 69.00 (7.00)	50% 47% 53%	Strength	65 min/sess., 3 x/w	682.5	Passive	Usual care	8 w	GDS
Sjösten et al., 2008 [65]	Finland	Aerobic + Therapy Control	293 298	32 29	72.70 (5.40) 72.80 (5.90)	76%	Aerobic + Therapy	2 x/m	-	Passive	Usual care	12 m	GDS
Tsang et al., 2006 [66]	China	Qigong Control	48 34	0 0	82.11 (7.19) 82.74 (6.83)	83%	Qigong	45 min/sess., 3 x/w	405	Passive	Educational	16 w	GDS
Underwood et al., 2013 [67]	The UK	Exercise Depression awareness training	174 200	51 64	86.6 (7.4) 86.7 (7.8)	75% 74%	Multisport	2 x/week	-	Active	Depression awareness training	24 w	GDS
Williams et al., 2008 [68]	USA	Aerobic + Strength Walking Control	16 17 12	1 6 0	87.90 (5.95)	89%	Aerobic + Strength Walking	30 min/sess., 5 x/w	1425 375	Passive	Social	16 w	CSDD

Abbreviation: CBT: Cognitive Behavioral Therapy; DIG: Delayed Intervention Group; HADS: Hospital Anxiety and Depression Scale; HIFE: High-Intensity Functional Exercise program; HIIT: High-Intensity Interval Training; HRSD: Hamilton Rating Scale for Depression; ICBT: Internet-Based Cognitive–Behavioral Therapy; IIG: Immediate Intervention Group; LMCM: Lifestyle Modification with Contingency Management; LMIEP: Low to Moderate Intensity Exercise Program; MET: Metabolic Equivalent of Task; MICEP: Moderate-Intensity Exercise Program; MICT: Moderate-to-Vigorous Intensity Continuous Training; OEP: Otago Exercise Program; PMR: Progressive Muscle Relaxation; SADQ: Stroke Aphasic Depression Questionnaire; TDQ: Taiwanese Depression Questionnaire; w: Weeks; m: Months; x: Times.

**Table 2:** Comparison and ranking of different exercise interventions aimed at improving depressive symptoms in older adults.

Qigong											−1.17 [−2.28; −0.06]	
−0.02 [−1.61; 1.57]	Otago Exercise										−1.15 [−2.29; −0.01]	
−0.29 [−1.59; 1.00]	−0.27 [−1.60; 1.06]	Yoga		−0.46 [−1.64; 0.72]						−0.13 [−1.17; 0.91]	−1.27 [−2.07; −0.46]	
−0.35 [−1.57; 0.88]	−0.32 [−1.58; 0.93]	−0.05 [−0.89; 0.78]	Walking		−0.17 [−1.29; 0.94]				−0.04 [−1.31; 1.23]		−0.82 [−1.41; −0.24]	
−0.43 [−1.98; 1.12]	−0.41 [−1.98; 1.16]	−0.14 [−1.21; 0.93]	−0.08 [−1.27; 1.10]	Jogging							−1.08 [−2.27; 0.12]	
−0.50 [−1.67; 0.67]	−0.47 [−1.68; 0.73]	−0.20 [−0.94; 0.53]	−0.15 [−0.73; 0.43]	−0.07 [−1.19; 1.06]	Strength					−0.87 [−1.69; −0.06]	−0.69 [−1.12; −0.26]	−0.66 [−1.71; 0.39]
−0.55 [−1.86; 0.77]	−0.53 [−1.87; 0.82]	−0.26 [−1.22; 0.71]	−0.20 [−1.06; 0.66]	−0.12 [−1.40; 1.17]	−0.05 [−0.84; 0.74]	Aerobic			−0.35 [−1.45; 0.74]		−0.71 [−1.46; 0.04]	
−0.63 [−2.18; 0.92]	−0.61 [−2.18; 0.96]	−0.34 [−1.61; 0.93]	−0.29 [−1.48; 0.91]	−0.20 [−1.73; 1.32]	−0.14 [−1.28; 1.01]	−0.08 [−1.37; 1.21]	Physical Training				−0.54 [−1.62; 0.54]	
−0.66 [−2.00; 0.69]	−0.64 [−2.01; 0.74]	−0.37 [−1.38; 0.65]	−0.31 [−1.23; 0.61]	−0.23 [−1.55; 1.09]	−0.16 [−1.00; 0.68]	−0.11 [−1.15; 0.93]	−0.03 [−1.35; 1.30]	Tai chi			−0.06 [−0.87; 0.76]	−3.99 [−6.06; −1.92]
−0.85 [−2.06; 0.35]	−0.83 [−2.07; 0.41]	−0.56 [−1.33; 0.21]	−0.51 [−1.16; 0.15]	−0.42 [−1.58; 0.74]	−0.36 [−0.91; 0.19]	−0.31 [−1.08; 0.47]	−0.22 [−1.40; 0.96]	−0.19 [−1.08; 0.69]	Multisport	−0.32 [−1.16; 0.51]	−0.30 [−0.88; 0.28]	−0.66 [−1.75; 0.44]
−1.07 [−2.33; 0.19]	−1.05 [−2.34; 0.24]	−0.78 [−1.51; −0.04]	−0.72 [−1.48; 0.03]	−0.64 [−1.81; 0.54]	−0.57 [−1.17; 0.02]	−0.52 [−1.42; 0.37]	−0.44 [−1.67; 0.80]	−0.41 [−1.37; 0.55]	−0.22 [−0.83; 0.40]	Control Active		
−1.17 [−2.28; −0.06]	−1.15 [−2.29; −0.01]	−0.88 [−1.55; −0.21]	−0.82 [−1.34; −0.31]	−0.74 [−1.82; 0.34]	−0.67 [−1.05; −0.30]	−0.62 [−1.33; 0.08]	−0.54 [−1.62; 0.54]	−0.51 [−1.28; 0.25]	−0.32 [−0.79; 0.16]	−0.10 [−0.70; 0.50]	Control Passive	
−1.72 [−3.07; −0.37]	−1.70 [−3.08; −0.32]	−1.43 [−2.42; −0.43]	−1.37 [−2.27; −0.47]	−1.29 [−2.60; 0.03]	−1.22 [−1.99; −0.46]	−1.17 [−2.19; −0.15]	−1.09 [−2.42; 0.24]	−1.06 [−2.08; −0.05]	−0.87 [−1.64; −0.09]	−0.65 [−1.55; 0.25]	−0.55 [−1.32; 0.23]	Control Waitlist

## Data Availability

The data are included in the article and the Supplementary Materials.

## Supplementary Materials

The following supporting information can be downloaded at: https://www.mdpi.com/article/10.3390/healthcare12151498/s1, Figure S1. Summary of the quality assessment for included studies. Figure S2. The forest plot of pairwise comparisons for different exercise interventions to improve depressive symptoms in elderly patients, retrieved from the included trials, demonstrates the standardized mean difference (SMD). Figure S3. The forest plot of pairwise comparisons for different exercise interventions to improve depressive symptoms in elderly patients, retrieved from the included trials, demonstrates the risk difference (RD) of dropout rates. None of the comparisons reached statistical significance. Figure S4. The forest plots display the results of the sensitivity analysis conducted using the one-study removal method, involving 31 studies (labeled 1 to 31). The ranking and clinical significance remain unchanged, indicating that the conclusions of our study are not affected by the inclusion or exclusion of any single study. Figure S5. Forest plot displaying the improvement in depressive symptoms in elderly patients after receiving different types of exercise interventions, presented as standardized mean differences (SMDs). The pre-post correlation coefficient used in the calculation of data was changed from 0.8 used in Figure 3 to 0.5 in this figure as a sensitivity analysis. The ranking and clinical interpretations remained unchanged compared to Figure 3. This suggests that the conclusions of our study remain consistent despite different assumptions regarding the coefficient used for transformation. Figure S6. Based on the Egger’s test results, the figure shows the following information: the intercept (B0) is 2.595, with a 95% confidence interval of (1.350, 3.841); the t-value is 4.263 with 29 degrees of freedom; the 1-tailed *p*-value (recommended) is 0.001, and the 2-tailed *p*-value is 0.001. These data indicate that the intercept value significantly deviates from zero, suggesting the presence of publication bias. Specifically, the very small *p*-value for the intercept shows statistical significance, further supporting the conclusion of bias. Table S1. PRISMA for network meta-analysis checklist. Table S2. Keywords and search results in different databases. Table S3. Studies excluded from the analysis along with the reasons for their exclusion. Table S4. Detailed quality assessment of included studies using Cochrane risk of bias 2 tool (RoB2). Table S5. Inconsistency test outcomes for the standardized mean difference in improving depressive symptoms in elderly patients treated with exercise interventions. Table S6. Inconsistency test results for the risk difference in dropout rates when applying exercise interventions to alleviate depressive symptoms in elderly patients.

## References

[1] World Health Organization (2023). Depressive Disorder (Depression).

[2] Tu C.Y., Liao S.C., Wu C.S., Chiu Y.T., Huang W.L. (2024). Association of categorical diagnoses and psychopathologies with quality of life in patients with depression, anxiety, and somatic symptoms: A cross-sectional study. J. Psychosom. Res..

[3] Marawi T., Zhukovsky P., Brooks H., Bowie C.R., Butters M.A., Fischer C.E., Flint A.J., Herrmann N., Lanctôt K.L., Mah L. (2024). Heterogeneity of Cognition in Older Adults with Remitted Major Depressive Disorder: A Latent Profile Analysis. Am. J. Geriatr. Psychiatry.

[4] Invernizzi S., Simoes Loureiro I., Kandana Arachchige K.G., Lefebvre L. (2021). Late-Life Depression, Cognitive Impairment, and Relationship with Alzheimer’s Disease. Dement. Geriatr. Cogn. Disord..

[5] Patrick R.E., Dickinson R.A., Gentry M.T., Kim J.U., Oberlin L.E., Park S., Principe J.L., Teixeira A.L., Weisenbach S.L. (2024). Treatment resistant late-life depression: A narrative review of psychosocial risk factors, non-pharmacological interventions, and the role of clinical phenotyping. J. Affect. Disord..

[6] Weng W.H., Wang Y.H., Yeh N.C., Yang Y.R., Wang R.Y. (2024). Effects of physical training on depression and related quality of life in pre-frail and frail older adults: A systematic review and meta-analysis. J. Nutr. Health Aging.

[7] Zhang Y., Jiang X. (2023). The effects of physical activity and exercise therapy on frail elderly depression: A narrative review. Medicine.

[8] Izquierdo M., Merchant R., Morley J., Anker S., Aprahamian I., Arai H., Aubertin-Leheudre M., Bernabei R., Cadore E., Cesari M. (2021). International exercise recommendations in older adults (ICFSR): Expert consensus guidelines. J. Nutr. Health Aging.

[9] Huang J., Wang D., Wang J. (2021). Clinical evidence of tai chi exercise prescriptions: A systematic review. Evid. Based Complement. Altern. Med..

[10] Wehner C., Blank C., Arvandi M., Wehner C., Schobersberger W. (2021). Effect of Tai Chi on muscle strength, physical endurance, postural balance and flexibility: A systematic review and meta-analysis. BMJ Open Sport Exerc. Med..

[11] Pascoe M.C., J de Manincor M., Hallgren M., Baldwin P.A., Tseberja J., Parker A.G. (2021). Psychobiological mechanisms underlying the mental health benefits of yoga-based interventions: A narrative review. Mindfulness.

[12] Yang Y., Wang K., Liu H., Qu J., Wang Y., Chen P., Zhang T., Luo J. (2022). The impact of Otago exercise programme on the prevention of falls in older adult: A systematic review. Front. Public Health.

[13] Carta M.G., Cossu G., Pintus E., Zoccheddu R., Callia O., Conti G., Pintus M., Gonzalez C.I.A., Massidda M.V., Mura G. (2021). Active elderly and health—Can moderate exercise improve health and wellbeing in older adults? Protocol for a randomized controlled trial. Trials.

[14] Bai X., Soh K.G., Omar Dev R.D., Talib O., Xiao W., Cai H. (2022). Effect of brisk walking on health-related physical fitness balance and life satisfaction among the elderly: A systematic review. Front. Public Health.

[15] Rodrigues J.M., Lopes L.T., Gonçalves M., Machado J.P. (2023). Perceived Health Benefits of Taijiquan and Qigong. Altern. Ther. Health Med..

[16] Wang R., Huang X., Wu Y., Sun D. (2021). Efficacy of qigong exercise for treatment of fatigue: A systematic review and meta-analysis. Front. Med..

[17] Wingood M., Bonnell L., LaCroix A.Z., Rosenberg D., Walker R., Bellettiere J., Greenwood-Hickman M.A., Wing D., Gell N. (2021). Community-dwelling older adults and physical activity recommendations: Patterns of aerobic, strengthening, and balance activities. J. Aging Phys. Act..

[18] Claudino J.G., Afonso J., Sarvestan J., Lanza M.B., Pennone J., Filho C.A.C., Serrão J.C., Espregueira-Mendes J., Vasconcelos A.L.V., de Andrade M.P. (2021). Strength training to prevent falls in older adults: A systematic review with meta-analysis of randomized controlled trials. J. Clin. Med..

[19] Yildiz A., Vieta E., Correll C.U., Nikodem M., Baldessarini R.J. (2014). Critical issues on the use of network meta-analysis in psychiatry. Harv. Rev. Psychiatry.

[20] Nevill C.R., Cooper N.J., Sutton A.J. (2023). A multifaceted graphical display, including treatment ranking, was developed to aid interpretation of network meta-analysis. J. Clin. Epidemiol..

[21] Chaimani A., Caldwell D.M., Li T., Higgins J.P., Salanti G. (2019). Chapter 11: Undertaking Network Meta-Analyses. Cochrane Handbook for Systematic Reviews of Interventions version 6.3. Cochrane Handbook for Systematic Reviews of Interventions.

[22] Su X., McDonough D.J., Chu H., Quan M., Gao Z. (2020). Application of network meta-analysis in the field of physical activity and health promotion. J. Sport. Health Sci..

[23] Hutton B., Salanti G., Caldwell D.M., Chaimani A., Schmid C.H., Cameron C., Ioannidis J.P., Straus S., Thorlund K., Jansen J.P. (2015). The PRISMA extension statement for reporting of systematic reviews incorporating network meta-analyses of health care interventions: Checklist and explanations. Ann. Intern. Med..

[24] Zhang J., Yuan Y., Chu H. (2016). The Impact of Excluding Trials from Network Meta-Analyses—An Empirical Study. PLoS ONE.

[25] Sterne J.A.C., Savović J., Page M.J., Elbers R.G., Blencowe N.S., Boutron I., Cates C.J., Cheng H.Y., Corbett M.S., Eldridge S.M. (2019). RoB 2: A revised tool for assessing risk of bias in randomised trials. BMJ.

[26] Greenberg S.A. (2012). The geriatric depression scale (GDS). Best Pract. Nurs. Care Older Adults.

[27] Jackson-Koku G. (2016). Beck depression inventory. Occup. Med..

[28] Bech P. (2009). Fifty years with the Hamilton scales for anxiety and depression: A tribute to Max Hamilton. Psychother. Psychosom..

[29] Lappan S.N., Brown A.W., Hendricks P.S. (2020). Dropout rates of in-person psychosocial substance use disorder treatments: A systematic review and meta-analysis. Addiction.

[30] Deeks J.J., Higgins J.P., Altman D.G., Group C.S.M. (2019). Analysing data and undertaking meta-analyses. Cochrane Handbook for Systematic Reviews of Interventions.

[31] Higgins J.P., Eldridge S., Li T. (2019). Including variants on randomized trials. Cochrane Handbook for Systematic Reviews of Interventions.

[32] Page M.J., Higgins J.P., Sterne J.A. (2019). Assessing risk of bias due to missing results in a synthesis. Cochrane Handbook for Systematic Reviews of Interventions.

[33] Borenstein M., Hedges L.V., Higgins J.P., Rothstein H.R. (2009). Fixed-effect versus random-effects models. Introd. Meta-Anal..

[34] Owen R.K., Bradbury N., Xin Y., Cooper N., Sutton A. (2019). MetaInsight: An interactive web-based tool for analyzing, interrogating, and visualizing network meta-analyses using R-shiny and netmeta. Res. Synth. Methods.

[35] Becker L.A. (2000). Effect Size (ES). https://www.uv.es/friasnav/EffectSizeBecker.pdf.

[36] Higgins J.P., Li T., Deeks J.J. (2019). Choosing effect measures and computing estimates of effect. Cochrane Handbook for Systematic Reviews of Interventions.

[37] Pearson M.J., Smart N.A. (2018). Reported methods for handling missing change standard deviations in meta-analyses of exercise therapy interventions in patients with heart failure: A systematic review. PLoS ONE.

[38] Aguiñaga S., Ehlers D.K., Salerno E.A., Fanning J., Motl R.W., McAuley E. (2018). Home-Based Physical Activity Program Improves Depression and Anxiety in Older Adults. J. Phys. Act. Health.

[39] Belvederi Murri M., Amore M., Menchetti M., Toni G., Neviani F., Cerri M., Rocchi M.B., Zocchi D., Bagnoli L., Tam E. (2015). Physical exercise for late-life major depression. Br. J. Psychiatry.

[40] Boström G., Conradsson M., Hörnsten C., Rosendahl E., Lindelöf N., Holmberg H., Nordström P., Gustafson Y., Littbrand H. (2016). Effects of a high-intensity functional exercise program on depressive symptoms among people with dementia in residential care: A randomized controlled trial. Int. J. Geriatr. Psychiatry.

[41] Brittle N., Patel S., Wright C., Baral S., Versfeld P., Sackley C. (2009). An exploratory cluster randomized controlled trial of group exercise on mobility and depression in care home residents. Clin. Rehabil..

[42] Chang K.J., Hong C.H., Roh H.W., Lee K.S., Lee E.H., Kim J., Lim H.K., Son S.J. (2018). A 12-Week Multi-Domain Lifestyle Modification to Reduce Depressive Symptoms in Older Adults: A Preliminary Report. Psychiatry Investig..

[43] Chen K.M., Chen M.H., Chao H.C., Hung H.M., Lin H.S., Li C.H. (2009). Sleep quality, depression state, and health status of older adults after silver yoga exercises: Cluster randomized trial. Int. J. Nurs. Stud..

[44] Chen H.M., Tsai C.M., Wu Y.C., Lin K.C., Lin C.C. (2015). Randomised controlled trial on the effectiveness of home-based walking exercise on anxiety, depression and cancer-related symptoms in patients with lung cancer. Br. J. Cancer.

[45] Chen X., Zhao L., Liu Y., Zhou Z., Zhang H., Wei D., Chen J., Li Y., Ou J., Huang J. (2021). Otago exercise programme for physical function and mental health among older adults with cognitive frailty during COVID-19: A randomised controlled trial. J. Clin. Nurs..

[46] Chou K.L., Lee P.W., Yu E.C., Macfarlane D., Cheng Y.H., Chan S.S., Chi I. (2004). Effect of Tai Chi on depressive symptoms amongst Chinese older patients with depressive disorders: A randomized clinical trial. Int. J. Geriatr. Psychiatry.

[47] Conradsson M., Littbrand H., Lindelof N., Gustafson Y., Rosendahl E. (2010). Effects of a high-intensity functional exercise programme on depressive symptoms and psychological well-being among older people living in residential care facilities: A cluster-randomized controlled trial. Aging Ment. Health.

[48] Cheng S.T., Chow P.K., Yu E.C., Chan A.C. (2012). Leisure activities alleviate depressive symptoms in nursing home residents with very mild or mild dementia. Am. J. Geriatr. Psychiatry.

[49] Danhauer S.C., Miller M.E., Divers J., Anderson A., Hargis G., Brenes G.A. (2022). Long-Term Effects of Cognitive-Behavioral Therapy and Yoga for Worried Older Adults. Am. J. Geriatr. Psychiatry.

[50] de Lima T.A., Ferreira-Moraes R., Alves W., Alves T.G.G., Pimentel C.P., Sousa E.C., Abrahin O., Cortinhas-Alves E.A. (2019). Resistance training reduces depressive symptoms in elderly people with Parkinson disease: A controlled randomized study. Scand. J. Med. Sci. Sports.

[51] Deus L.A., Corrêa H.L., Neves R.V.P., Reis A.L., Honorato F.S., Silva V.L., Souza M.K., de Araújo T.B., de Gusmão Alves L.S., Sousa C.V. (2021). Are Resistance Training-Induced BDNF in Hemodialysis Patients Associated with Depressive Symptoms, Quality of Life, Antioxidant Capacity, and Muscle Strength? An Insight for the Muscle-Brain-Renal Axis. Int. J. Environ. Res. Public Health.

[52] Gary R.A., Dunbar S.B., Higgins M.K., Musselman D.L., Smith A.L. (2010). Combined exercise and cognitive behavioral therapy improves outcomes in patients with heart failure. J. Psychosom. Res..

[53] Huang T.T., Liu C.B., Tsai Y.H., Chin Y.F., Wong C.H. (2015). Physical fitness exercise versus cognitive behavior therapy on reducing the depressive symptoms among community-dwelling elderly adults: A randomized controlled trial. Int. J. Nurs. Stud..

[54] Lavretsky H., Milillo M.M., Kilpatrick L., Grzenda A., Wu P., Nguyen S.A., Ercoli L.M., Siddarth P. (2022). A Randomized Controlled Trial of Tai Chi Chih or Health Education for Geriatric Depression. Am. J. Geriatr. Psychiatry.

[55] Lok N., Lok S., Canbaz M. (2017). The effect of physical activity on depressive symptoms and quality of life among elderly nursing home residents: Randomized controlled trial. Arch. Gerontol. Geriatr..

[56] Makizako H., Tsutsumimoto K., Doi T., Makino K., Nakakubo S., Liu-Ambrose T., Shimada H. (2019). Exercise and Horticultural Programs for Older Adults with Depressive Symptoms and Memory Problems: A Randomized Controlled Trial. J. Clin. Med..

[57] McMurdo M.E., Burnett L. (1992). Randomised controlled trial of exercise in the elderly. Gerontology.

[58] Moraes H.S., Silveira H.S., Oliveira N.A., Matta Mello Portugal E., Araújo N.B., Vasques P.E., Bergland A., Santos T.M., Engedal K., Coutinho E.S. (2020). Is Strength Training as Effective as Aerobic Training for Depression in Older Adults? A Randomized Controlled Trial. Neuropsychobiology.

[59] Ng T.P., Nyunt M.S.Z., Feng L., Feng L., Niti M., Tan B.Y., Chan G., Khoo S.A., Chan S.M., Yap P. (2017). Multi-Domains Lifestyle Interventions Reduces Depressive Symptoms among Frail and Pre-Frail Older Persons: Randomized Controlled Trial. J. Nutr. Health Aging.

[60] Seino S., Nishi M., Murayama H., Narita M., Yokoyama Y., Nofuji Y., Taniguchi Y., Amano H., Kitamura A., Shinkai S. (2017). Effects of a multifactorial intervention comprising resistance exercise, nutritional and psychosocial programs on frailty and functional health in community-dwelling older adults: A randomized, controlled, cross-over trial. Geriatr. Gerontol. Int..

[61] Shahidi M., Mojtahed A., Modabbernia A., Mojtahed M., Shafiabady A., Delavar A., Honari H. (2011). Laughter yoga versus group exercise program in elderly depressed women: A randomized controlled trial. Int. J. Geriatr. Psychiatry.

[62] Shahtahmassebi B., Hatton J., Hebert J.J., Hecimovich M., Correia H., Fairchild T.J. (2022). The effect of the inclusion of trunk-strengthening exercises to a multimodal exercise program on physical activity levels and psychological functioning in older adults: Secondary data analysis of a randomized controlled trial. BMC Geriatr..

[63] Singh N.A., Clements K.M., Fiatarone M.A. (1997). A randomized controlled trial of progressive resistance training in depressed elders. J. Gerontol. A Biol. Sci. Med. Sci..

[64] Singh N.A., Stavrinos T.M., Scarbek Y., Galambos G., Liber C., Fiatarone Singh M.A. (2005). A randomized controlled trial of high versus low intensity weight training versus general practitioner care for clinical depression in older adults. J. Gerontol. A Biol. Sci. Med. Sci..

[65] Sjösten N.M., Vahlberg T.J., Kivelä S.L. (2008). The effects of multifactorial fall prevention on depressive symptoms among the aged at increased risk of falling. Int. J. Geriatr. Psychiatry.

[66] Tsang H.W., Fung K.M., Chan A.S., Lee G., Chan F. (2006). Effect of a qigong exercise programme on elderly with depression. Int. J. Geriatr. Psychiatry.

[67] Underwood M., Lamb S.E., Eldridge S., Sheehan B., Slowther A., Spencer A., Thorogood M., Atherton N., Bremner S.A., Devine A. (2013). Exercise for depression in care home residents: A randomised controlled trial with cost-effectiveness analysis (OPERA). Health Technol. Assess..

[68] Williams C.L., Tappen R.M. (2008). Exercise training for depressed older adults with Alzheimer’s disease. Aging Ment. Health.

[69] Tang L., Zhang L., Liu Y., Li Y., Yang L., Zou M., Yang H., Zhu L., Du R., Shen Y. (2024). Optimal dose and type of exercise to improve depressive symptoms in older adults: A systematic review and network meta-analysis. BMC Geriatr..

[70] Correia É.M., Monteiro D., Bento T., Rodrigues F., Cid L., Vitorino A., Figueiredo N., Teixeira D.S., Couto N. (2024). Analysis of the Effect of Different Physical Exercise Protocols on Depression in Adults: Systematic Review and Meta-analysis of Randomized Controlled Trials. Sports Health.

[71] Mahmoudi A., Amirshaghaghi F., Aminzadeh R., Mohamadi Turkmani E. (2022). Effect of Aerobic, Resistance, and Combined Exercise Training on Depressive Symptoms, Quality of Life, and Muscle Strength in Healthy Older Adults: A Systematic Review and Meta-Analysis of Randomized Controlled Trials. Biol. Res. Nurs..

[72] Piva G., Caruso L., Gómez A.C., Calzolari M., Visintin E.P., Davoli P., Manfredini F., Storari A., Spinozzi P., Lamberti N. (2024). Effects of forest walking on physical and mental health in elderly populations: A systematic review. Rev. Environ. Health.

[73] Milton-Cole R., Kazeem K., Gibson A., Guerra S., Sheehan K.J. (2024). Effectiveness of exercise rehabilitation interventions on depressive symptoms in older adults post hip fracture: A systematic review and meta-analysis. Osteoporos. Int..

[74] Cohen-Mansfield J., Marx M.S., Biddison J.R., Guralnik J.M. (2004). Socio-environmental exercise preferences among older adults. Prev. Med..

[75] Silva L.A.d., Tortelli L., Motta J., Menguer L., Mariano S., Tasca G., Silveira G.d.B., Pinho R.A., Silveira P.C.L. (2019). Effects of aquatic exercise on mental health, functional autonomy and oxidative stress in depressed elderly individuals: A randomized clinical trial. Clinics.

[76] Yang P.-Y., Ho K.-H., Chen H.-C., Chien M.-Y. (2012). Exercise training improves sleep quality in middle-aged and older adults with sleep problems: A systematic review. J. Physiother..

[77] Liu F., Cui J., Liu X., Chen K.W., Chen X., Li R. (2020). The effect of tai chi and Qigong exercise on depression and anxiety of individuals with substance use disorders: A systematic review and meta-analysis. BMC Complement. Med. Ther..

[78] Voss S., Cerna J., Gothe N.P. (2023). Yoga impacts cognitive health: Neurophysiological changes and stress regulation mechanisms. Exerc. Sport Sci. Rev..

[79] Aditi Devi N., Phillip M., Varambally S., Christopher R., Gangadhar B.N. (2023). Yoga as a monotherapy alters proBDNF—Mature BDNF ratio in patients with major depressive disorder. Asian J. Psychiatr..

[80] Kim I., Ahn J. (2021). The effect of changes in physical self-concept through participation in exercise on changes in self-esteem and mental well-being. Int. J. Environ. Res. Public Health.

[81] Craft L.L. (2023). Potential psychological mechanisms underlying the exercise and depression relationship. Routledge Handbook of Physical Activity and Mental Health.

[82] Abdulrasool M.D., EmadOdaJoda A., Abdulrasool M.D. (2020). The effect of psycho-physiological sports proposed in terms of the hormone endorphins serotonin and their relative results on mental fitness in the aged. Ann. Trop. Med. Public Health.

[83] Latino F., Tafuri F. (2024). Physical Activity and Cognitive Functioning. Medicina.

[84] Kazeminia M., Salari N., Vaisi-Raygani A., Jalali R., Abdi A., Mohammadi M., Daneshkhah A., Hosseinian-Far M., Shohaimi S. (2020). The effect of exercise on anxiety in the elderly worldwide: A systematic review and meta-analysis. Health Qual. Life Outcomes.

[85] Ruiz-Comellas A., Valmaña G.S., Catalina Q.M., Baena I.G., Mendioroz Peña J., Roura Poch P., Sabata Carrera A., Cornet Pujol I., Casaldàliga Solà À., Fusté Gamisans M. (2022). Effects of physical activity interventions in the elderly with anxiety, depression, and low social support: A clinical multicentre randomised trial. Healthcare.

[86] Zhang Y., Su D., Chen Y., Tan M., Chen X. (2022). Effect of socioeconomic status on the physical and mental health of the elderly: The mediating effect of social participation. BMC Public Health.

[87] Weyerer S., Kupfer B. (1994). Physical exercise and psychological health. Sports Med..

[88] Langoni C.d.S., Resende T.d.L., Barcellos A.B., Cecchele B., da Rosa J.N., Knob M.S., Silva T.d.N., Diogo T.d.S., da Silva I.G., Schwanke C.H.A. (2019). The effect of group exercises on balance, mobility, and depressive symptoms in older adults with mild cognitive impairment: A randomized controlled trial. Clin. Rehabil..

[89] Singh N.A., Clements K.M., Singh M.A.F. (2001). The efficacy of exercise as a long-term antidepressant in elderly subjects: A randomized, controlled trial. J. Gerontol. Ser. A Biol. Sci. Med. Sci..

