# Supplementary material for: Motivational Barriers and Facilitators for Older Adults’ Engagement in Exercise during Depressive Episodes: A Network Meta-Analysis of Randomized Controlled Trials
Source: Healthcare (Basel). 2024 Jul 28;12(15):1498. doi: 10.3390/healthcare12151498 (PMC11311502; doi:10.3390/healthcare12151498)
Supplement: Supplementary file 1 [file healthcare-12-01498-s001.zip › healthcare-3100509-supplementary.pdf]

Table S1 - PRISMA for network meta-analysis checklist

Section and Topic	#	Checklist item	Location
Title			
Title	1	Identify the report as a systematic review incorporating a network meta-analysis (or related form of meta-analysis).	Title
Abstract			
Structured summary	2	Provide a structured summary including, as applicable: Background: main objectives / Methods: data sources; study eligibility criteria, participants, and interventions; study appraisal; and synthesis methods, such as network meta-analysis. / Results: number of studies and participants identified; summary estimates with corresponding confidence/credible intervals; treatment rankings may also be discussed. Authors may choose to summarize pairwise comparisons against a chosen treatment included in their analyses for brevity. / Discussion/conclusions: limitations; conclusions and implications of findings. / Other: primary source of funding; systematic review registration number with registry name.	Abstract
Introduction			
Rationale	3	Describe the rationale for the review in the context of what is already known, including mention of why a network meta-analysis has been conducted.	Introduction / 1st and 2nd paragraph
Objectives	4	Provide an explicit statement of questions being addressed, with reference to participants, interventions, comparisons, outcomes, and study design (PICOS).	Introduction / 3rd paragraph
Methods			
Protocol & registration	5	Indicate whether a review protocol exists and where it can be accessed; and, if available, provide registration information, including registration number.	Materials and Methods/ 1st paragrph
Eligibility criteria	6	Specify study characteristics (e.g., PICOS, length of follow-up) and report characteristics (e.g., years considered, language, publication status) used as criteria for eligibility, giving rationale. Clearly describe eligible treatments included in the treatment network, and note whether any have been clustered or merged into the same node (with justification).	Materials and Methods/ Inculusion and exculusion
Information sources	7	Describe all information sources (e.g., databases with dates of coverage, contact with study authors) in the search and date last searched.	Table S2
Search	8	Present full electronic search strategy for at least one database, including any limits used, such that it could be repeated.	Table S2
Study selection	9	State the process for selecting studies (i.e., screening, eligibility, included in systematic review, and, if applicable, included in the meta-analysis).	Materials and Methods / Study Identification / Inculusion and exculusion criteria
Data collection	10	Describe method of data extraction from reports (e.g., piloted forms, independently, in duplicate) and any processes for obtaining and confirming data.	Materials and Methods / Data extraction
Data items	11	List and define all variables for which data were sought (e.g., PICOS, funding sources) and any assumptions and simplifications made.	Materials and Methods / Data extraction and conversion
Network geometry	S1	Describe methods used to explore the geometry of the treatment network under study and potential biases related to it. This should include how the evidence base has been graphically summarized for presentation, and what characteristics were compiled and used to describe the evidence base to readers.	Materials and Methods / Modeling for network meta-analysis
Risk of bias within	12	Describe methods used for assessing risk of bias of individual studies (including specification of whether this was done at the study or outcome level), and how this information is to be used in any data synthesis.	Materials and Methods / Quality appraisal
Summary measures	13	State the principal summary measures (e.g., risk ratio, difference in means). Also describe the use of additional summary measures assessed, such as treatment rankings, as well as modified approaches used to present summary findings from meta-analyses.	Materials and Methods / Outcome
Planned methods of analysis	14	Describe the methods of handling data and combining results of studies for each network meta-analysis. This should include, but not be limited to: Handling of multi-arm trials; Selection of variance structure; Selection of prior distributions in Bayesian analyses; and Assessment of model fit.	Materials and Methods / Statistical analyses
Assessment of	S2	Describe the statistical methods used to evaluate the agreement of direct and indirect evidence in	Materials and Methods

inconsistency		the treatment network(s) studied. Describe efforts taken to address its presence when found.	/ Statistical analyses
Risk of bias across	15	Specify any assessment of risk of bias that may affect the cumulative evidence.	Materials and Methods / Publication bias
Additional analyses	16	Describe methods of additional analyses if done, indicating which were pre-specified. This may include, but not be limited to, the following: Sensitivity or subgroup analyses; Meta-regression analyses; Alternative formulations of the treatment network; and Use of alternative prior distributions for Bayesian analyses (if applicable).	Materials and Methods / Sensitivity analyses
Results			
Study selection	17	Give numbers of studies screened, assessed for eligibility, and included in the review, with reasons for exclusions at each stage, ideally with a flow diagram.	Results / Study identification Figure 1, Table S2, Table S3
Network structure	S3	Provide a network graph of the included studies to enable visualization of the geometry of the treatment network.	Fugre 2
Network geometry	S4	Provide a brief overview of characteristics of the treatment network. This may include commentary on the abundance of trials and randomized patients for the different interventions and pairwise comparisons in the network, gaps of evidence in the treatment network, and potential biases reflected by the network structure.	Results / Network model formation / Figure 2
Study characteristics	18	For each study, present characteristics for which data were extracted (e.g., study size, PICOS, follow-up period) and provide the citations.	Table 1
Risk of bias within	19	Present data on risk of bias of each study and, if available, any outcome level assessment.	Table S4, Figure S1, Methodological quality
Results of individual studies	20	For all outcomes considered (benefits or harms), present, for each study: (1) simple summary data for each intervention group, and (2) effect estimates and confidence intervals. Modified approaches may be needed to deal with information from larger networks.	Table 1
Synthesis of results	21	Present results of each meta-analysis done, including confidence/credible intervals. In larger networks, authors may focus on comparisons versus a particular comparator (e.g. placebo or standard care), with full findings presented in an appendix. League tables and forest plots may be considered to summarize pairwise comparisons. If additional summary measures were explored (such as treatment rankings), these should also be presented.	Outcomes / Figure 3, Figure 4, Figure S2, Figure S3, Table 2
Exploration for inconsistency	S5	Describe results from investigations of inconsistency. This may include such information as measures of model fit to compare consistency and inconsistency models, P values from statistical tests, or summary of inconsistency estimates from different parts of the treatment network.	Inconsistency test Table S5, Table S6
Risk of bias across	22	Present results of any assessment of risk of bias across studies for the evidence base being studied.	Publicaton bias, Figure S6
Additional analyses	23	Give results of additional analyses, if done (e.g., sensitivity or subgroup analyses, meta-regression analyses, alternative network geometries studied, alternative choice of prior distributions for Bayesian analyses, and so forth).	Sensitivity analysis / Figure S4, Figre S5
Discussion			
Summary of evidence	24	Summarize the main findings, including the strength of evidence for each main outcome; consider their relevance to key groups.	Discussion Findings and implications
Limitations	25	Discuss limitations at study and outcome level (e.g., risk of bias), and at review level (e.g., incomplete retrieval of identified research, reporting bias). Comment on the validity of the assumptions, such as transitivity and consistency. Comment on any concerns regarding network geometry (e.g., avoidance of certain comparisons).	Discussion Linitations
Conclusions	26	Provide a general interpretation of the results in the context of other evidence, and implications for future research.	Conclusion
Funding			
Funding	27	Describe sources of funding for the systematic review and other support (e.g., supply of data); role of funders for the systematic review. This should also include information regarding whether funding has been received from manufacturers of treatments in the network and/or whether some of the authors are content experts with professional conflicts of interest that could affect use of treatments in the network.	Funding

PICOS, population, intervention, comparators, outcomes, study design.

Table S2 - Keywords and search results in different databases

Database	Keyword	Date	Results
PubMed	('aerobic' OR 'aquatic exercise' OR 'jogging' OR 'otago' OR 'qi-gong' OR 'strength' OR 'tai-chi' OR 'walking' OR 'yoga') AND ('exercise') AND ('depression') AND ('elderly' OR 'geriatric') AND ('random' OR 'randomized' OR 'randomised')	2024.04.30	3395
Embase	('aerobic' OR 'aquatic exercise' OR 'jogging' OR 'otago' OR 'qi-gong' OR 'strength' OR 'tai-chi' OR 'walking' OR 'yoga') AND ('exercise') AND ('depression') AND ('elderly' OR 'geriatric') AND ('random' OR 'randomized' OR 'randomised')	2024.04.30	1037
Cochrane CENTRAL	('aerobic' OR 'aquatic exercise' OR 'jogging' OR 'otago' OR 'qi-gong' OR 'strength' OR 'tai-chi' OR 'walking' OR 'yoga') AND ('exercise') AND ('depression') AND ('elderly' OR 'geriatric') AND ('random' OR 'randomized' OR 'randomised')	2024.04.30	504
Web of Science	('aerobic' OR 'aquatic exercise' OR 'jogging' OR 'otago' OR 'qi-gong' OR 'strength' OR 'tai-chi' OR 'walking' OR 'yoga') AND ('exercise') AND ('depression') AND ('elderly' OR 'geriatric') AND ('random' OR 'randomized' OR 'randomised')	2024.04.30	269
			5205

Table S3 - Studies excluded from the analysis along with the reasons for their exclusion

Authors & year / Title		Journal/Book	Exclusion reasons
Apóstolo 2019	Effectiveness of a Combined Intervention on Psychological and Physical Capacities of Frail Older Adults: A Cluster Randomized Controlled Trial	Int J Environ Res Public Health	Control with other intervention
Bernard 2015	Exercise and Counseling for Smoking Cessation in Smokers With Depressive Symptoms: A Randomized Controlled Pilot Trial	J Dual Diagn.	Unsuitable population
Bouaziz 2019	Effects of a short-term Interval Aerobic Training Programme with active Recovery bouts (IATP-R) on cognitive and mental health, functional performance and quality of life: A randomised controlled trial in sedentary seniors	Int J Clin Pract.	No relevant outcome data
Brenes 2007	Treatment of minor depression in older adults: a pilot study comparing sertraline and exercise	Aging Ment Health.	Pilot study
Chalder 2012	Facilitated physical activity as a treatment for depressed adults: randomised controlled trial	BMJ.	Irrelevant intervention
Chen 2020	Efficacy of multidomain interventions to improve physical frailty, depression and cognition: data from cluster-randomized controlled trials	J Cachexia Sarcopenia Muscle.	No relevant outcome data
Chen 2023	Effects of Resistance Exercise on Cognitive Performance and Depressive Symptoms in Community-Dwelling Older Chinese Americans: A Pilot Randomized Controlled Trial	Behav Sci (Basel).	Pilot study
Chin 2022	Effects of Exercise Frequency and Intensity on Reducing Depressive Symptoms in Older Adults With Insomnia: A Pilot Randomized Controlled Trial	Front Physiol.	Pilot study
Choi 2020	Effect of Telehealth Treatment by Lay Counselors vs by Clinicians on Depressive Symptoms Among Older Adults Who Are Homebound: A Randomized Clinical Trial	JAMA Netw Open	Irrelevant intervention
D'Amato 1990	The relative efficacy of structured reminiscence and physical exercise on clinically depressed geriatric inpatients	ProQuest / Hofstra University.	Incomplete Data
Forsyth 2015	A lifestyle intervention for primary care patients with depression and anxiety: A randomised controlled trial	Psychiatry Res.	Irrelevant intervention
Heissel 2015	Feasibility of an exercise program for older depressive inpatients: A pilot study.	GeroPsych	Pilot study
Helgadóttir 2017	Long-term effects of exercise at different intensity levels on depression: A randomized controlled trial	Prev Med.	No relevant outcome data
Helgadóttir B	Training fast or slow? Exercise for depression: A randomized controlled trial	Prev Med.	Unsuitable population
Ho 2020	Psychophysiological Effects of Dance Movement Therapy and Physical Exercise on Older Adults With Mild Dementia: A Randomized Controlled Trial	J Gerontol B Psychol Sci Soc Sci.	No relevant outcome data
Isaksen 2016	Effects of aerobic interval training on measures of anxiety, depression and quality of life in patients with ischaemic heart failure and an implantable cardioverter defibrillator: A prospective non-randomized trial	J Rehabil Med.	Not RCT
Krogh 2007	Design paper: the DEMO trial: a randomized, parallel-group, observer-blinded clinical trial of aerobic versus non-aerobic versus relaxation training for patients with light to moderate depression	Contemp Clin Trials.	Protocol
Krogh 2009	The DEMO trial: a randomized, parallel-group, observer-blinded clinical trial of strength versus aerobic versus relaxation training for patients with mild to moderate depression	J Clin Psychiatry.	Unsuitable population
Krogh 2014	The effect of exercise on hippocampal volume and neurotrophines in patients with major depression--a randomized clinical trial	J Affect Disord	Incomplete Data
Lanza 2019	Shiatsu as an adjuvant therapy for depression in patients with Alzheimer's disease: A pilot study	J Evid Based Integr Med.	Pilot study
Legrand 2009	The effects of 60 minutes of supervised weekly walking (in a single vs. 3-5 session format) on depressive symptoms among older women: findings from a pilot randomized trial	Mental Health and Physical Activity	Pilot study
Liao 2018	The Impact of Combined Music and Tai Chi on Depressive Symptoms Among Community-Dwelling Older Persons: a Cluster Randomized Controlled Trial	Issues Ment Health Nurs	Incomplete Data
Mather 2002	Effects of exercise on depressive symptoms in older adults with poorly responsive depressive disorder : randomised controlled trial	Br J Psychiatry.	Unsuitable population
Matthews 2020	Effects of a multicomponent exercise program combined with calcium-vitamin D(3)-enriched milk on health-related quality of life and depressive symptoms in older men: secondary analysis of a randomized controlled trial	Eur J Nutr	Incomplete Data
Minghetti 2018	Sprint interval training (SIT) substantially reduces depressive symptoms in major depressive disorder (MDD): A randomized controlled trial	Psychiatry Res.	Incomplete Data

Mitchell 2008	Living well with stroke: design and methods for a randomized controlled trial of a psychosocial behavioral intervention for poststroke depression	J Stroke Cerebrovasc Dis.	Irrelevant intervention
Prathikanti 2017	Treating major depression with yoga: A prospective, randomized, controlled pilot trial	PLoS One.	Pilot study
Pynnönen 2018	Effect of a social intervention of choice vs. control on depressive symptoms, melancholy, feeling of loneliness, and perceived togetherness in older Finnish people: a randomized controlled trial	Aging Ment Health.	No relevant outcome data
Silva 2019	Effects of aquatic exercise on mental health, functional autonomy and oxidative stress in depressed elderly individuals: A randomized clinical trial	Clinics (Sao Paulo).	Unsuitable population
Singh 1996	A randomised controlled trial of exercise in depressed elders	Nurs Open	Incomplete Data
Singh 2001	The efficacy of exercise as a long-term antidepressant in elderly subjects: a randomized, controlled trial	J Gerontol A Biol Sci Med Sci.	Secondary Analysis
Stahl 2020	Digital Monitoring of Sleep, Meals, and Physical Activity for Reducing Depression in Older Spousally-Bereaved Adults: A Pilot Randomized Controlled Trial	Am J Geriatr Psychiatry	Pilot study
Trivedi 2011	Exercise as an augmentation treatment for nonremitted major depressive disorder: a randomized, parallel dose comparison	J Clin Psychiatry.	Incomplete Data
Uebelacker 2017	Adjunctive yoga v. health education for persistent major depression: a randomized controlled trial	Psychol Med.	Incomplete Data
Uebelacker LA	Treatment credibility, expectancy, and preference: Prediction of treatment engagement and outcome in a randomized clinical trial of hatha yoga vs. health education as adjunct treatments for depression	J Affect Disord.	Secondary Analysis
Verrusio 2014	Exercise training and music therapy in elderly with depressive syndrome: a pilot study	Complementary therapies in medicine	Pilot study
Vestergaard 2023	Impact of prestroke physical activity and citalopram treatment on poststroke depressive symptoms: a secondary analysis of data from the TALOS randomised controlled trial in Denmark	BMJ Open	No relevant outcome data
von Berens 2018	Effect of exercise and nutritional supplementation on health-related quality of life and mood in older adults: the VIVE2 randomized controlled trial	BMC Geriatr	Control with other intervention
Walker 2010	Mental health literacy, folic acid and vitamin B12, and physical activity for the prevention of depression in older adults: randomised controlled trial	Br J Psychiatry	Incomplete Data
Yilmaz 2019	Effect of progressive muscle relaxation on the caregiver burden and level of depression among caregivers of older patients with a stroke: A randomized controlled trial	Jpn J Nurs Sci.	Unsuitable population
Yu 2023	Comparison of moderate and vigorous walking exercise on reducing depression in middle-aged and older adults: A pilot randomized controlled trial	Eur J Sport Sci	Pilot study
Zou 2005	Interventional efficacy of citalopram combined with shining and psychological morning exercise in the attack of depression in elderly people	Chin J Clin Rehabil	Incomplete Data
Abdelbasset 2019	Similar effects of low to moderate-intensity exercise program vs moderate-intensity continuous exercise program on depressive disorder in heart failure patients: A 12-week randomized controlled trial	Medicine (Baltimore)	Unsuitable population
Aidar 2018	A randomized trial of the effects of an aquatic exercise program on depression, anxiety levels, and functional capacity of people who suffered an ischemic stroke	J Sports Med Phys Fitness.	Unsuitable population
Hallgren 2015	Physical exercise and internet-based cognitive-behavioural therapy in the treatment of depression: randomised controlled trial	Br J Psychiatry.	Unsuitable population
Martiny 2012	A 9-week randomized trial comparing a chronotherapeutic intervention (wake and light therapy) to exercise in major depressive disorder patients treated with duloxetine	J Clin Psychiatry.	Unsuitable population
Reed 2022	The effects of high-intensity interval training, Nordic walking and moderate-to-vigorous intensity continuous training on functional capacity, depression and quality of life in patients with coronary artery disease enrolled in cardiac rehabilitation: A randomized controlled trial (CRX study)	Prog Cardiovasc Dis.	Unsuitable population
Veale 1992	Aerobic exercise in the adjunctive treatment of depression: a randomized controlled trial	J R Soc Med.	Unsuitable population

Table S4 – Detailed quality assessment of included studies using Cochrane risk of bias 2 tool (RoB2)

First author & Year	Randomization process	Intervention adherence	Missing outcome data	Outcome measurement	Selective reporting	Overall RoB
Aguinaga et al., 2018	L	L3	L	L	L	L
Belvederi Murri et al., 2015	L	L3	L	L	L	L
Boström et al., 2016	L	L3	L	L	L	L
Brittle et al., 2009	L	L3	L	L	L	L
Chang et al., 2018	L	L2	L	S	L	S
Chen et al., 2009	L	L1	L	S	L	S
Chen et al., 2015	L	L3	L	L	L	L
Chen et al., 2021	L	L3	L	L	L	L
Cheng et al., 2012	L	L2	L	S	L	S
Chou et al., 2004	L	L1	L	S	L	S
Conradsson et al., 2010	L	L3	L	L	L	L
Danhauer et al., 2022	L	L3	L	L	L	L
de Lima et al., 2019	L	L3	L	L	L	L
Deus et al., 2021	L	L3	L	L	L	L
Gary et al., 2010	L	S2	L	S	L	S
Huang et al., 2015	L	L3	L	L	L	L
Lavretsky et al., 2022	L	L3	L	L	L	L
Lok et al., 2017	L	L3	L	L	L	L
Makizako et al., 2020	L	S2	L	S	L	S
McMurdo et al., 2001	L	L3	L	L	L	L
Moraes et al., 2020	L	L3	L	L	L	L
Ng et al., 2017	L	S2	L	S	L	S
Seino et al., 2017	L	L3	L	L	L	L
Shahidi et al., 2011	L	L3	L	L	L	L
Shahtahmassebi et al., 2022	L	S2	L	S	L	S
Singh et al., 1997	L	L3	L	L	L	L
Singh et al., 2005	L	L3	L	L	L	L
Sjosten et al., 2008	L	S2	L	S	L	S
Tsang et al., 2006	L	S2	L	S	L	S
Underwood et al., 2013	L	S2	L	S	L	S
Williams et al., 2008	L	L3	L	L	L	L

1 The study employed a waitlist control group design, which resulted in a more balanced comparison among different groups.

2 The differences in protocols among various groups may affect adherence and outcome.

3 Both groups were randomized to receive exercise interventions, and the study design utilized a balanced protocol, which minimized the impact on adherence.

H, high risk of bias; L, low risk of bias; S, some risk of bias.

Table S5 - Inconsistency test outcomes for the standardized mean difference in improving depressive symptoms in elderly patients treated with exercise interventions

Comparison	No.Studies	NMA	Direct	Indirect	Difference	95CI_lower	95CI_upper	p-Value
Aerobic:Control_Active	0.00	-0.52	NA	-0.52	NA	NA	NA	NA
Aerobic:Control_Passive	2.00	-0.62	-0.71	0.11	-0.82	-3.11	1.47	0.48
Aerobic:Control_Waitlist	0.00	-1.17	NA	-1.17	NA	NA	NA	NA
Aerobic:Jogging	0.00	0.12	NA	0.12	NA	NA	NA	NA
Aerobic:Multisport	1.00	-0.31	-0.35	-0.26	-0.10	-1.65	1.45	0.90
Aerobic:Otago Exercise	0.00	0.53	NA	0.53	NA	NA	NA	NA
Aerobic:Physical Training	0.00	-0.08	NA	-0.08	NA	NA	NA	NA
Aerobic:Qigong	0.00	0.55	NA	0.55	NA	NA	NA	NA
Aerobic:Strength	0.00	0.05	NA	0.05	NA	NA	NA	NA
Aerobic:Tai-chi	0.00	-0.11	NA	-0.11	NA	NA	NA	NA
Aerobic:Walking	0.00	0.20	NA	0.20	NA	NA	NA	NA
Aerobic:Yoga	0.00	0.26	NA	0.26	NA	NA	NA	NA
Control_Active:Control_Passive	0.00	-0.10	NA	-0.10	NA	NA	NA	NA
Control_Active:Control_Waitlist	0.00	-0.65	NA	-0.65	NA	NA	NA	NA
Control_Active:Jogging	0.00	0.64	NA	0.64	NA	NA	NA	NA
Control_Active:Multisport	2.00	0.22	0.32	0.09	0.23	-1.00	1.46	0.71
Control_Active:Otago Exercise	0.00	1.05	NA	1.05	NA	NA	NA	NA
Control_Active:Physical Training	0.00	0.44	NA	0.44	NA	NA	NA	NA
Control_Active:Qigong	0.00	1.07	NA	1.07	NA	NA	NA	NA
Control_Active:Strength	2.00	0.57	0.87	0.23	0.64	-0.55	1.83	0.29
Control_Active:Tai-chi	0.00	0.41	NA	0.41	NA	NA	NA	NA
Control_Active:Walking	0.00	0.72	NA	0.72	NA	NA	NA	NA
Control_Active:Yoga	1.00	0.78	0.13	1.44	-1.31	-2.79	0.16	0.08
Control_Waitlist:Control_Passive	0.00	0.55	NA	0.55	NA	NA	NA	NA
Jogging:Control_Passive	1.00	-0.74	-1.08	0.75	-1.82	-4.60	0.95	0.20
Multisport:Control_Passive	4.00	-0.32	-0.30	-0.35	0.05	-0.96	1.07	0.92
Otago Exercise:Control_Passive	1.00	-1.15	-1.15	NA	NA	NA	NA	NA
Physical Training:Control_Passive	1.00	-0.54	-0.54	NA	NA	NA	NA	NA
Qigong:Control_Passive	1.00	-1.17	-1.17	NA	NA	NA	NA	NA
Strength:Control_Passive	7.00	-0.67	-0.69	-0.63	-0.06	-0.93	0.82	0.90
Tai-chi:Control_Passive	2.00	-0.51	-0.06	-3.91	3.86	1.48	6.23	0.00
Walking:Control_Passive	4.00	-0.82	-0.82	-0.83	0.01	-1.22	1.24	0.99
Yoga:Control_Passive	2.00	-0.88	-1.27	0.05	-1.31	-2.79	0.16	0.08
Control_Waitlist:Jogging	0.00	1.29	NA	1.29	NA	NA	NA	NA
Control_Waitlist:Multisport	1.00	0.87	0.66	1.08	-0.42	-1.98	1.13	0.59
Control_Waitlist:Otago Exercise	0.00	1.70	NA	1.70	NA	NA	NA	NA
Control_Waitlist:Physical Training	0.00	1.09	NA	1.09	NA	NA	NA	NA
Control_Waitlist:Qigong	0.00	1.72	NA	1.72	NA	NA	NA	NA
Control_Waitlist:Strength	1.00	1.22	0.66	1.85	-1.19	-2.72	0.34	0.13
Control_Waitlist:Tai-chi	1.00	1.06	3.99	0.14	3.86	1.48	6.23	0.00
Control_Waitlist:Walking	0.00	1.37	NA	1.37	NA	NA	NA	NA
Control_Waitlist:Yoga	0.00	1.43	NA	1.43	NA	NA	NA	NA
Jogging:Multisport	0.00	-0.42	NA	-0.42	NA	NA	NA	NA
Jogging:Otago Exercise	0.00	0.41	NA	0.41	NA	NA	NA	NA
Jogging:Physical Training	0.00	-0.20	NA	-0.20	NA	NA	NA	NA

Jogging:Qigong	0.00	0.43	NA	0.43	NA	NA	NA	NA
Jogging:Strength	0.00	-0.07	NA	-0.07	NA	NA	NA	NA
Jogging:Tai-chi	0.00	-0.23	NA	-0.23	NA	NA	NA	NA
Jogging:Walking	0.00	0.08	NA	0.08	NA	NA	NA	NA
Jogging:Yoga	1.00	0.14	0.46	-1.40	1.86	-0.97	4.69	0.20
Multisport:Otago Exercise	0.00	0.83	NA	0.83	NA	NA	NA	NA
Multisport:Physical Training	0.00	0.22	NA	0.22	NA	NA	NA	NA
Multisport:Qigong	0.00	0.85	NA	0.85	NA	NA	NA	NA
Multisport:Strength	0.00	0.36	NA	0.36	NA	NA	NA	NA
Multisport:Tai-chi	0.00	0.19	NA	0.19	NA	NA	NA	NA
Multisport:Walking	1.00	0.51	0.04	0.68	-0.63	-2.11	0.85	0.40
Multisport:Yoga	0.00	0.56	NA	0.56	NA	NA	NA	NA
Otago Exercise:Physical Training	0.00	-0.61	NA	-0.61	NA	NA	NA	NA
Otago Exercise:Qigong	0.00	0.02	NA	0.02	NA	NA	NA	NA
Otago Exercise:Strength	0.00	-0.47	NA	-0.47	NA	NA	NA	NA
Otago Exercise:Tai-chi	0.00	-0.64	NA	-0.64	NA	NA	NA	NA
Otago Exercise:Walking	0.00	-0.32	NA	-0.32	NA	NA	NA	NA
Otago Exercise:Yoga	0.00	-0.27	NA	-0.27	NA	NA	NA	NA
Physical Training:Qigong	0.00	0.63	NA	0.63	NA	NA	NA	NA
Physical Training:Strength	0.00	0.14	NA	0.14	NA	NA	NA	NA
Physical Training:Tai-chi	0.00	-0.03	NA	-0.03	NA	NA	NA	NA
Physical Training:Walking	0.00	0.29	NA	0.29	NA	NA	NA	NA
Physical Training:Yoga	0.00	0.34	NA	0.34	NA	NA	NA	NA
Qigong:Strength	0.00	-0.50	NA	-0.50	NA	NA	NA	NA
Qigong:Tai-chi	0.00	-0.66	NA	-0.66	NA	NA	NA	NA
Qigong:Walking	0.00	-0.35	NA	-0.35	NA	NA	NA	NA
Qigong:Yoga	0.00	-0.29	NA	-0.29	NA	NA	NA	NA
Strength:Tai-chi	0.00	-0.16	NA	-0.16	NA	NA	NA	NA
Strength:Walking	1.00	0.15	0.17	0.14	0.03	-1.28	1.34	0.96
Strength:Yoga	0.00	0.20	NA	0.20	NA	NA	NA	NA
Tai-chi:Walking	0.00	0.31	NA	0.31	NA	NA	NA	NA
Tai-chi:Yoga	0.00	0.37	NA	0.37	NA	NA	NA	NA
Walking:Yoga	0.00	0.05	NA	0.05	NA	NA	NA	NA

95CI-L: lower limit of 95% confidence interval; 95CI-U: upper limit of 95% confidence interval; NMA: network meta-analysis.

Table S6 - Inconsistency test results for the risk difference in dropout rates when applying exercise interventions to alleviate depressive symptoms in elderly patients.

Comparison	No.Studies	NMA	Direct	Indirect	Difference	95CI_lower	95CI_upper	p-Value
Aerobic:Control_Active	0.00	0.00	NA	0.00	NA	NA	NA	NA
Aerobic:Control_Passive	2.00	0.01	0.01	0.01	-0.01	-0.12	0.11	0.91
Aerobic:Control_Waitlist	0.00	-0.01	NA	-0.01	NA	NA	NA	NA
Aerobic:Jogging	0.00	0.00	NA	0.00	NA	NA	NA	NA
Aerobic:Multisport	1.00	0.00	0.00	0.00	0.00	-0.08	0.07	0.93
Aerobic:Otago Exercise	0.00	0.01	NA	0.01	NA	NA	NA	NA
Aerobic:Physical Training	0.00	0.01	NA	0.01	NA	NA	NA	NA
Aerobic:Qi-gong	0.00	0.01	NA	0.01	NA	NA	NA	NA
Aerobic:Strength	0.00	0.00	NA	0.00	NA	NA	NA	NA
Aerobic:Tai-chi	0.00	0.00	NA	0.00	NA	NA	NA	NA
Aerobic:Walking	0.00	-0.01	NA	-0.01	NA	NA	NA	NA
Aerobic:Yoga	0.00	0.00	NA	0.00	NA	NA	NA	NA
Control_Active:Control_Passive	0.00	0.01	NA	0.01	NA	NA	NA	NA
Control_Active:Control_Waitlist	0.00	-0.01	NA	-0.01	NA	NA	NA	NA
Control_Active:Jogging	0.00	0.00	NA	0.00	NA	NA	NA	NA
Control_Active:Multisport	1.00	0.00	0.03	0.00	0.03	-0.07	0.13	0.56
Control_Active:Otago Exercise	0.00	0.01	NA	0.01	NA	NA	NA	NA
Control_Active:Physical Training	0.00	0.01	NA	0.01	NA	NA	NA	NA
Control_Active:Qi-gong	0.00	0.01	NA	0.01	NA	NA	NA	NA
Control_Active:Strength	2.00	0.00	0.00	0.01	-0.01	-0.08	0.05	0.68
Control_Active:Tai-chi	0.00	0.00	NA	0.00	NA	NA	NA	NA
Control_Active:Walking	0.00	-0.01	NA	-0.01	NA	NA	NA	NA
Control_Active:Yoga	1.00	0.00	0.00	0.00	0.00	-0.07	0.08	0.98
Control_Waitlist:Control_Passive	0.00	0.02	NA	0.02	NA	NA	NA	NA
Jogging:Control_Passive	1.00	0.00	0.00	0.02	-0.02	-0.22	0.18	0.88
Multisport:Control_Passive	5.00	0.00	0.00	0.02	-0.02	-0.09	0.06	0.66
Otago Exercise:Control_Passive	1.00	0.00	0.00	NA	NA	NA	NA	NA
Physical Training:Control_Passive	1.00	0.00	0.00	NA	NA	NA	NA	NA
Qi-gong:Control_Passive	1.00	0.00	0.00	NA	NA	NA	NA	NA
Strength:Control_Passive	7.00	0.01	0.01	0.00	0.01	-0.05	0.07	0.81
Tai-chi:Control_Passive	2.00	0.01	0.01	0.02	-0.01	-0.28	0.25	0.91
Walking:Control_Passive	4.00	0.01	0.02	0.00	0.02	-0.08	0.11	0.70
Yoga:Control_Passive	2.00	0.01	0.01	0.01	0.00	-0.07	0.08	0.98
Control_Waitlist:Jogging	0.00	0.02	NA	0.02	NA	NA	NA	NA
Control_Waitlist:Multisport	1.00	0.02	0.00	0.10	-0.10	-0.22	0.02	0.10
Control_Waitlist:Otago Exercise	0.00	0.02	NA	0.02	NA	NA	NA	NA
Control_Waitlist:Physical Training	0.00	0.02	NA	0.02	NA	NA	NA	NA
Control_Waitlist:Qi-gong	0.00	0.02	NA	0.02	NA	NA	NA	NA
Control_Waitlist:Strength	1.00	0.02	0.11	-0.01	0.12	-0.01	0.25	0.07
Control_Waitlist:Tai-chi	1.00	0.01	0.00	0.01	-0.01	-0.28	0.25	0.91
Control_Waitlist:Walking	0.00	0.01	NA	0.01	NA	NA	NA	NA
Control_Waitlist:Yoga	0.00	0.01	NA	0.01	NA	NA	NA	NA
Jogging:Multisport	0.00	0.00	NA	0.00	NA	NA	NA	NA
Jogging:Otago Exercise	0.00	0.00	NA	0.00	NA	NA	NA	NA
Jogging:Physical Training	0.00	0.00	NA	0.00	NA	NA	NA	NA

Jogging:Qi-gong	0.00	0.00	NA	0.00	NA	NA	NA	NA
Jogging:Strength	0.00	0.00	NA	0.00	NA	NA	NA	NA
Jogging:Tai-chi	0.00	0.00	NA	0.00	NA	NA	NA	NA
Jogging:Walking	0.00	-0.01	NA	-0.01	NA	NA	NA	NA
Jogging:Yoga	1.00	0.00	0.00	-0.02	0.02	-0.18	0.22	0.88
Multisport:Otago Exercise	0.00	0.00	NA	0.00	NA	NA	NA	NA
Multisport:Physical Training	0.00	0.00	NA	0.00	NA	NA	NA	NA
Multisport:Qi-gong	0.00	0.00	NA	0.00	NA	NA	NA	NA
Multisport:Strength	0.00	0.00	NA	0.00	NA	NA	NA	NA
Multisport:Tai-chi	0.00	0.00	NA	0.00	NA	NA	NA	NA
Multisport:Walking	1.00	-0.01	-0.29	0.01	-0.30	-0.56	-0.03	0.03
Multisport:Yoga	0.00	0.00	NA	0.00	NA	NA	NA	NA
Otago Exercise:Physical Training	0.00	0.00	NA	0.00	NA	NA	NA	NA
Otago Exercise:Qi-gong	0.00	0.00	NA	0.00	NA	NA	NA	NA
Otago Exercise:Strength	0.00	-0.01	NA	-0.01	NA	NA	NA	NA
Otago Exercise:Tai-chi	0.00	-0.01	NA	-0.01	NA	NA	NA	NA
Otago Exercise:Walking	0.00	-0.01	NA	-0.01	NA	NA	NA	NA
Otago Exercise:Yoga	0.00	-0.01	NA	-0.01	NA	NA	NA	NA
Physical Training:Qi-gong	0.00	0.00	NA	0.00	NA	NA	NA	NA
Physical Training:Strength	0.00	-0.01	NA	-0.01	NA	NA	NA	NA
Physical Training:Tai-chi	0.00	-0.01	NA	-0.01	NA	NA	NA	NA
Physical Training:Walking	0.00	-0.01	NA	-0.01	NA	NA	NA	NA
Physical Training:Yoga	0.00	-0.01	NA	-0.01	NA	NA	NA	NA
Qi-gong:Strength	0.00	-0.01	NA	-0.01	NA	NA	NA	NA
Qi-gong:Tai-chi	0.00	-0.01	NA	-0.01	NA	NA	NA	NA
Qi-gong:Walking	0.00	-0.01	NA	-0.01	NA	NA	NA	NA
Qi-gong:Yoga	0.00	-0.01	NA	-0.01	NA	NA	NA	NA
Strength:Tai-chi	0.00	0.00	NA	0.00	NA	NA	NA	NA
Strength:Walking	1.00	-0.01	0.00	-0.02	0.02	-0.08	0.11	0.70
Strength:Yoga	0.00	0.00	NA	0.00	NA	NA	NA	NA
Tai-chi:Walking	0.00	0.00	NA	0.00	NA	NA	NA	NA
Tai-chi:Yoga	0.00	0.00	NA	0.00	NA	NA	NA	NA
Walking:Yoga	0.00	0.01	NA	0.01	NA	NA	NA	NA

95CI-L: lower limit of 95% confidence interval; 95CI-U: upper limit of 95% confidence interval; NMA: network meta-analysis.

Figure S1 - Summary of the quality assessment for included studies.

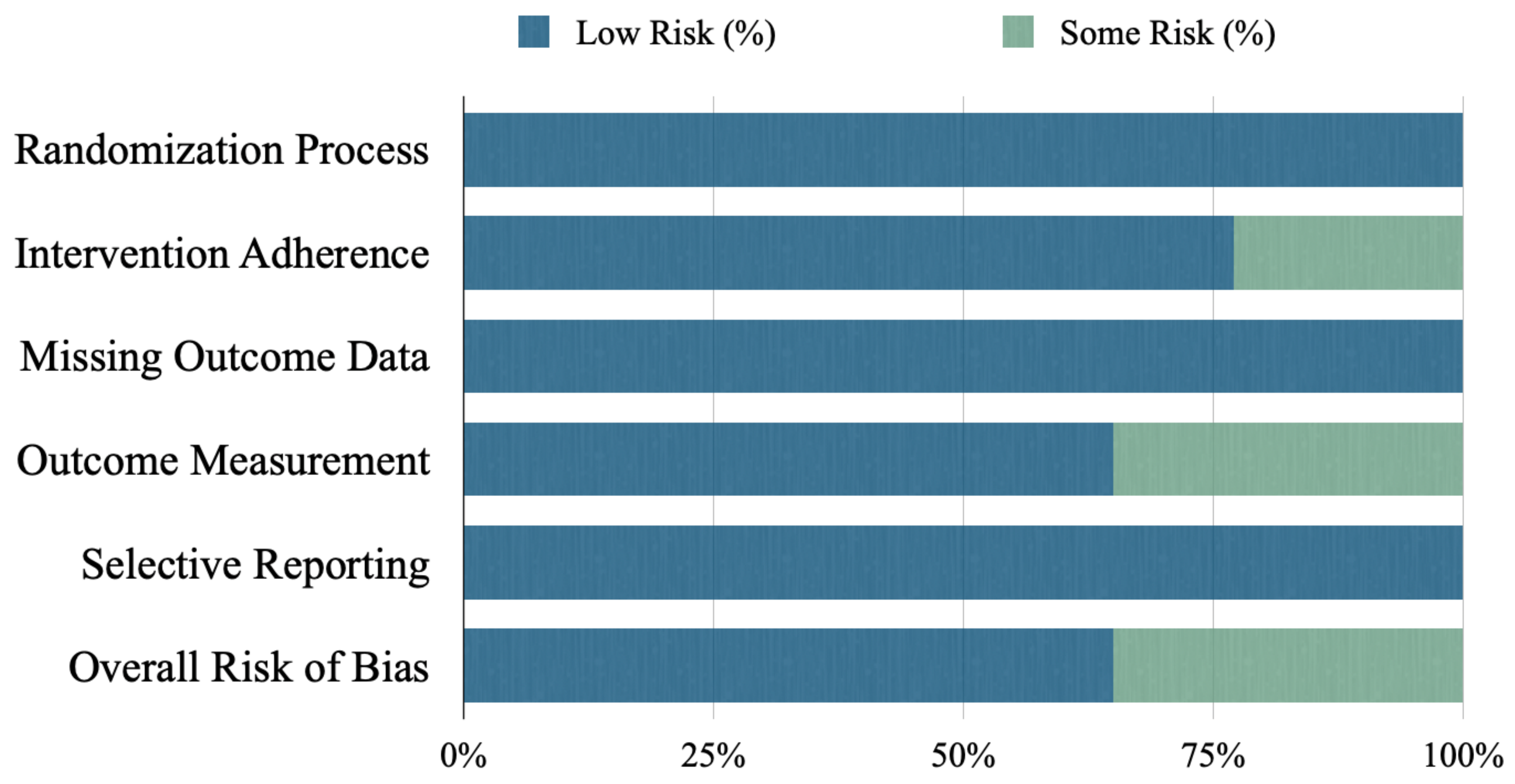

Analysis of the methodological quality across the 31 studies revealed the following specifics: In the randomization process, all studies showed low risk of bias, with 100% (31/31) maintaining stringent randomization standards. Intervention adherence varied, with 77% (24/31) demonstrating low risk and 23% (7/31) showing some risk, indicating a need for improved adherence protocols in a portion of the studies. Missing outcome data was impeccably managed in all studies, as evidenced by 100% (31/31) scoring low risk, which enhances the credibility of the study results. Outcome measurement was generally reliable with 65% (20/31) at low risk and 35% (11/31) at some risk, suggesting some studies could benefit from more meticulous measurement strategies. Selective reporting was excellently handled, with all studies transparently reporting outcomes, resulting in 100% (31/31) at low risk. The overall risk of bias was moderately concerning, with 65% (20/31) categorized as low risk and 35% (11/31) as some risk. Despite the generally low risk in most categories, the varying results in intervention adherence and overall risk of bias highlight areas where certain studies could enhance their methodological rigor. Detailed risk evaluations for each category can be referred to in Table S4.

Figure S2 - Individual study results (with studies excluded) grouped by treatment comparison

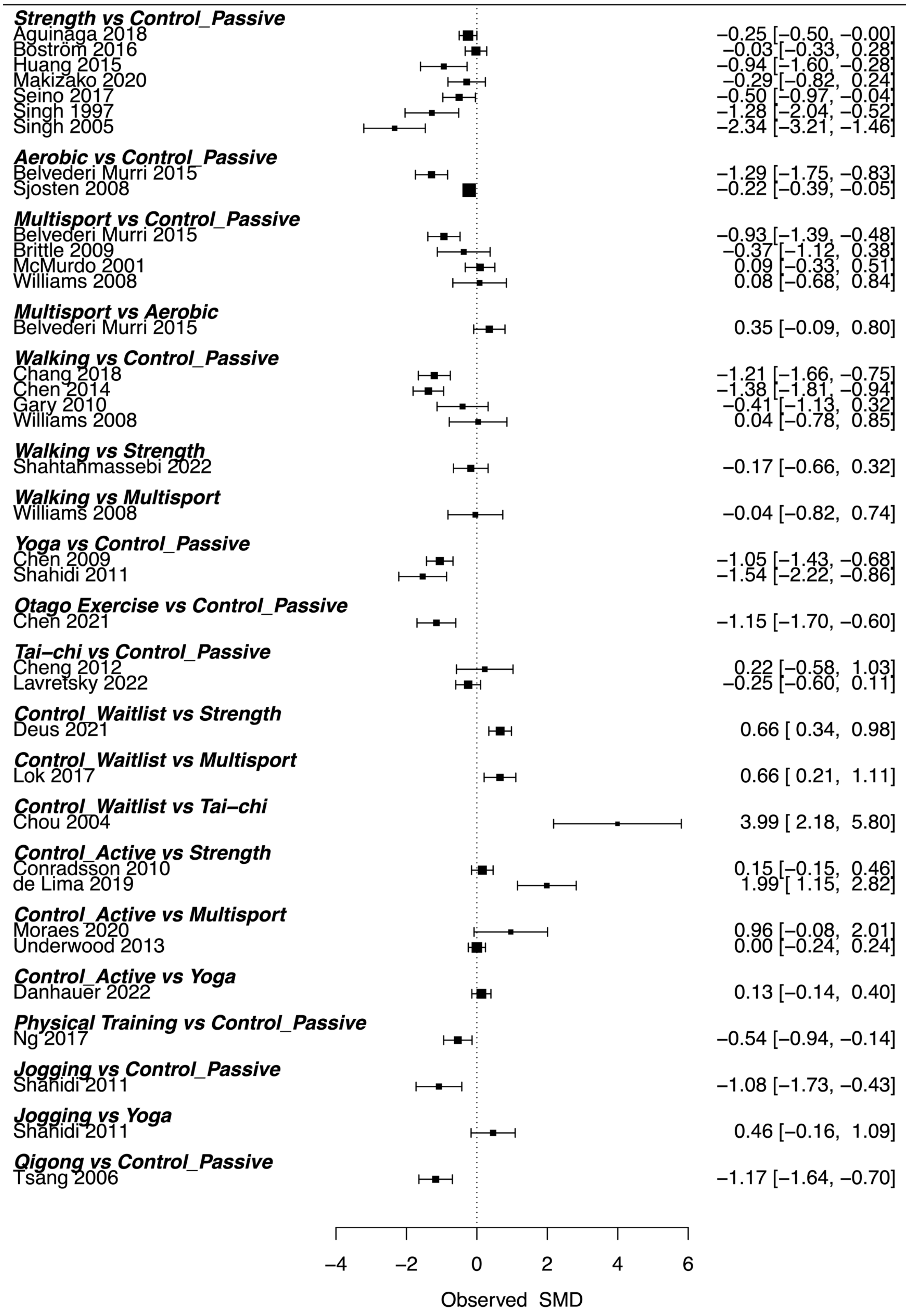

Fig. S2 - The forest plot of pairwise comparisons for different exercise interventions to improve depressive symptoms in elderly patients, retrieved from the included trials, demonstrates the standardized mean difference (SMD).

Figure S3 - Individual study results (with studies excluded) grouped by treatment comparison.

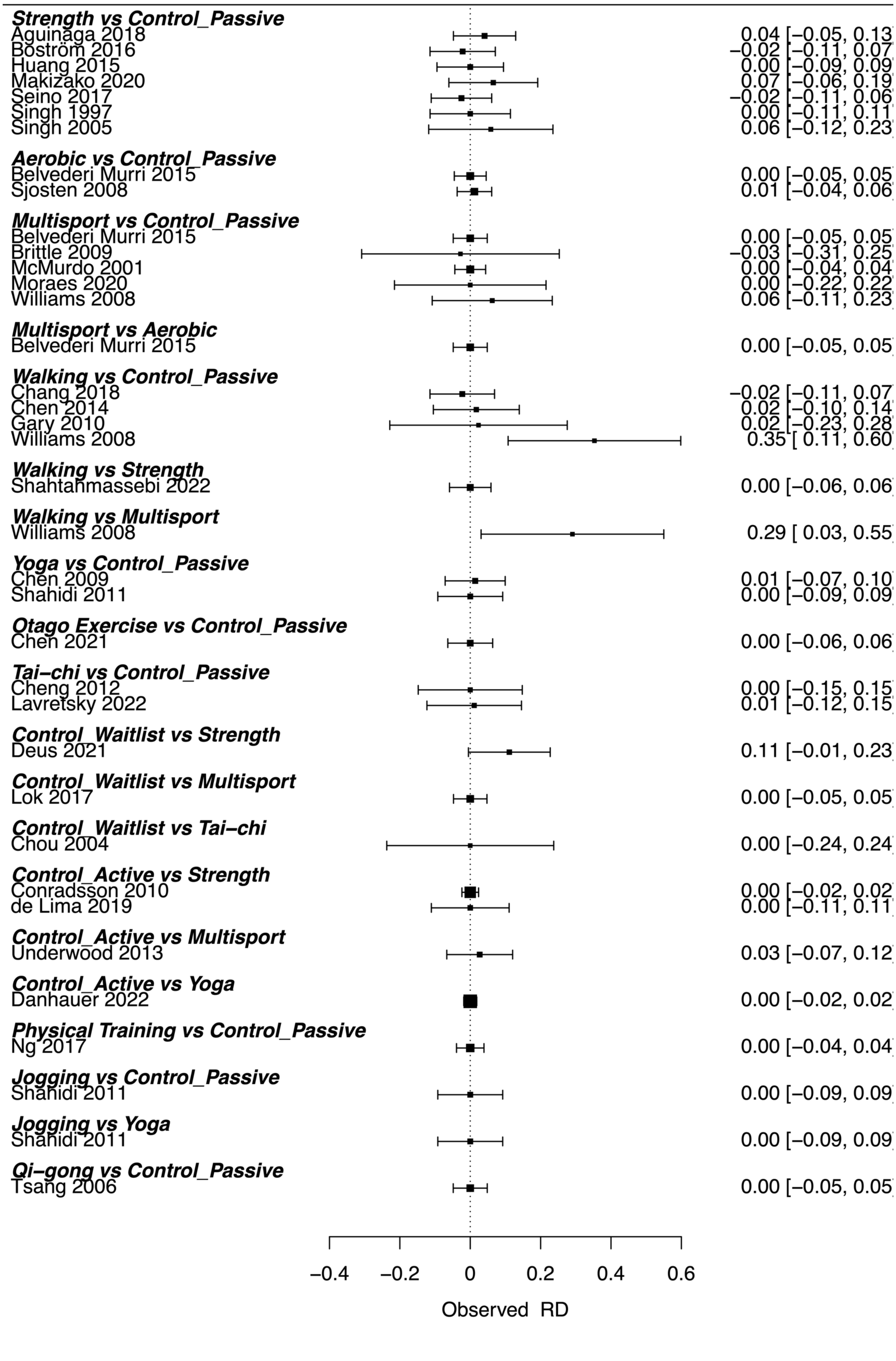

Fig. S3 - The forest plot of pairwise comparisons for different exercise interventions to improve depressive symptoms in elderly patients, retrieved from the included trials, demonstrates the risk difference (RD) of dropout rates. None of the comparisons reached statistical significance.

Fig. S4 -The forest plots display the results of the sensitivity analysis

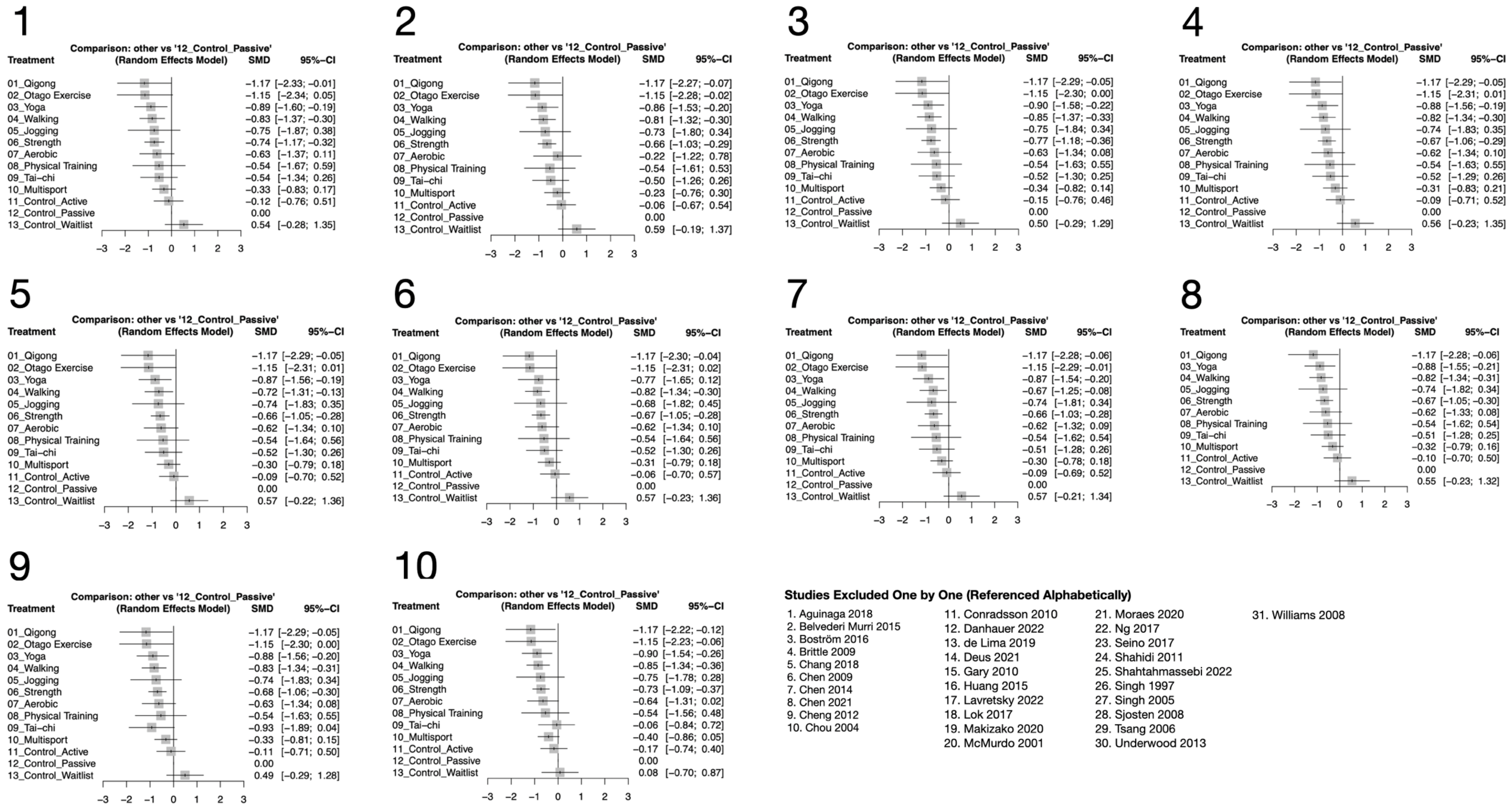

Fig. S4 -The forest plots display the results of the sensitivity analysis conducted using the one-study removal method, involving 31 studies (labeled 1 to 31). The ranking and clinical significance remain unchanged, indicating that the conclusions of our study are not affected by the inclusion or exclusion of any single study.

11

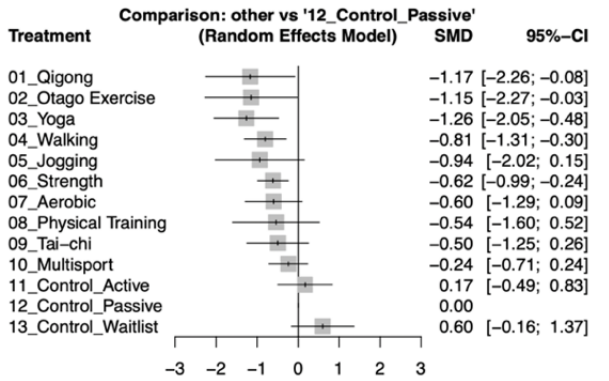

12

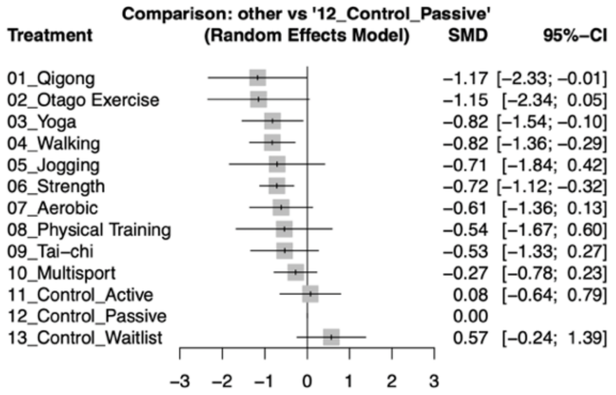

13

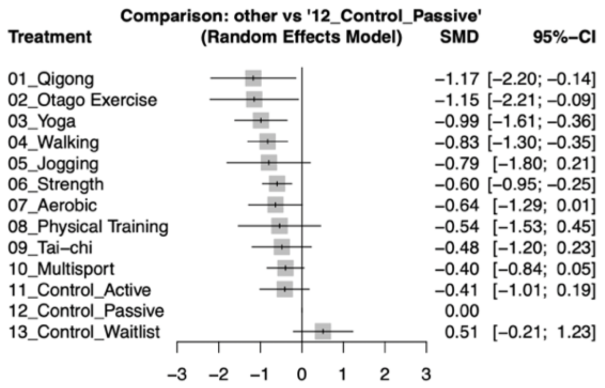

14

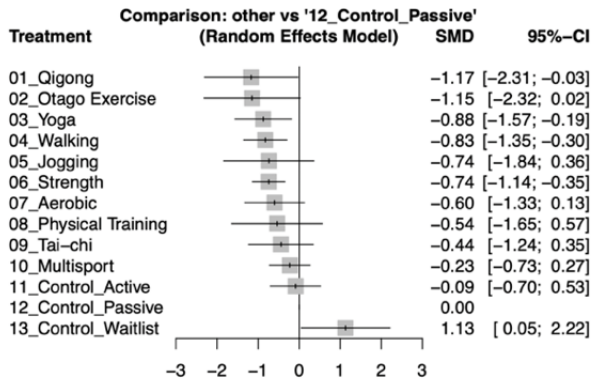

15

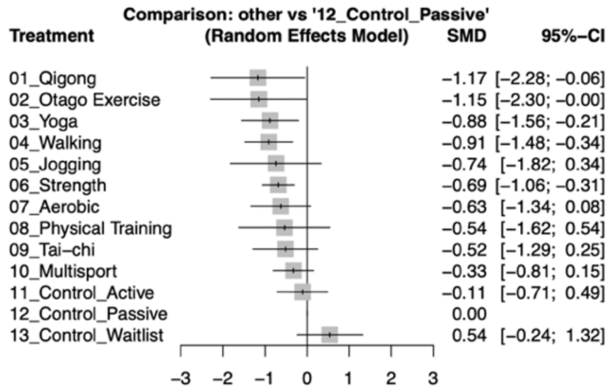

16

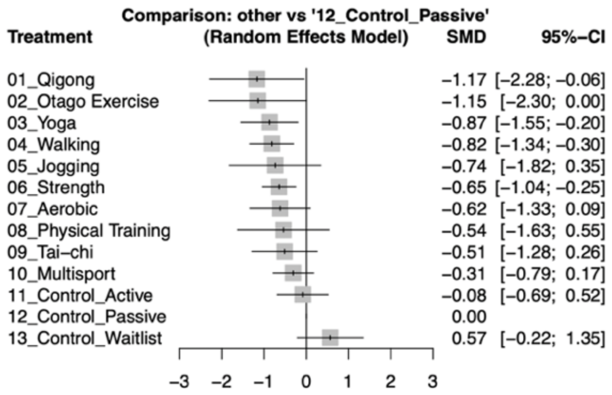

17

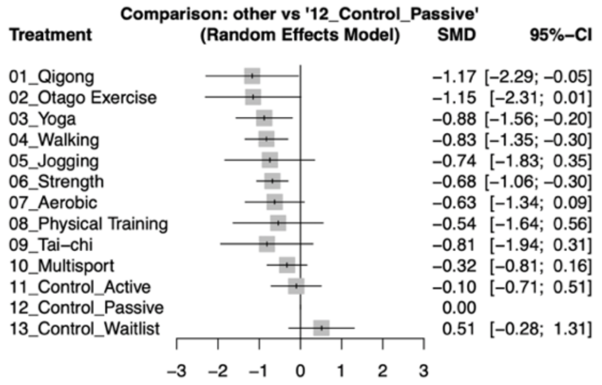

18

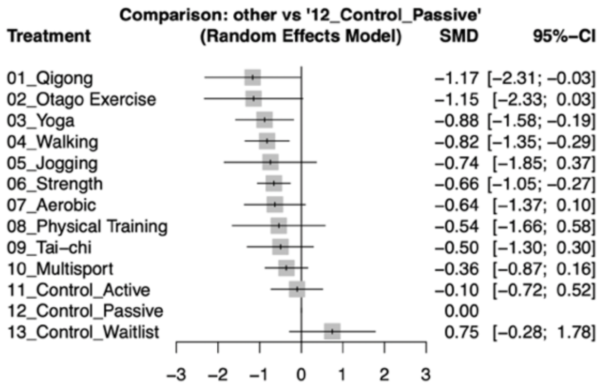

19

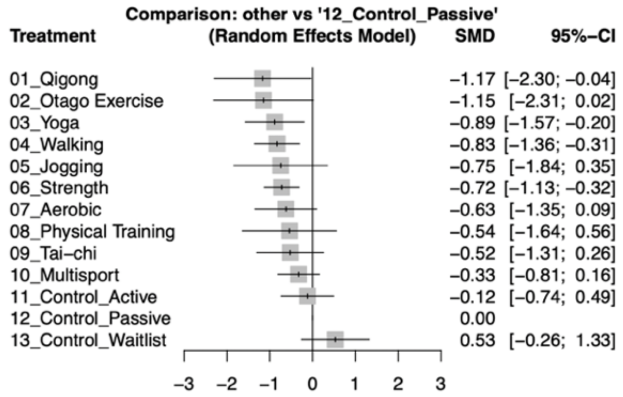

20

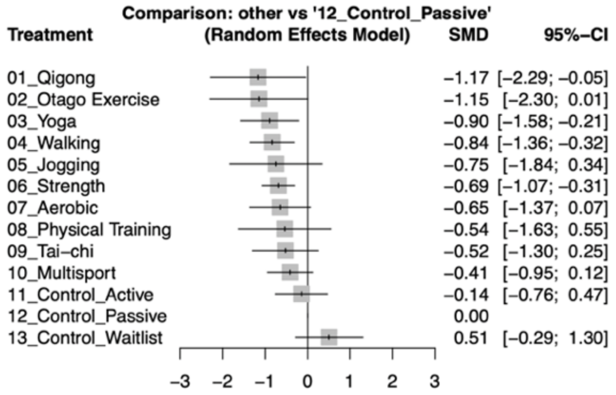

Studies Excluded One by One (Referenced Alphabetically)

1. Aguinaga 2018
2. Belvederi Murri 2015
3. Boström 2016
4. Brittle 2009
5. Chang 2018
6. Chen 2009
7. Chen 2014
8. Chen 2021
9. Cheng 2012
10. Chou 2004
11. Conradsson 2010
12. Danhauer 2022
13. de Lima 2019
14. Deus 2021
15. Gary 2010
16. Huang 2015
17. Lavretsky 2022
18. Lok 2017
19. Makizako 2020
20. McMurdo 2001
21. Moraes 2020
22. Ng 2017
23. Seino 2017
24. Shahidi 2011
25. Shahtahmassebi 2022
26. Singh 1997
27. Singh 2005
28. Sjosten 2008
29. Tsang 2006
30. Underwood 2013
31. Williams 2008

Fig. S4 -The forest plots display the results of the sensitivity analysis conducted using the one-study removal method, involving 31 studies (labeled 1 to 31). The ranking and clinical significance remain unchanged, indicating that the conclusions of our study are not affected by the inclusion or exclusion of any single study.

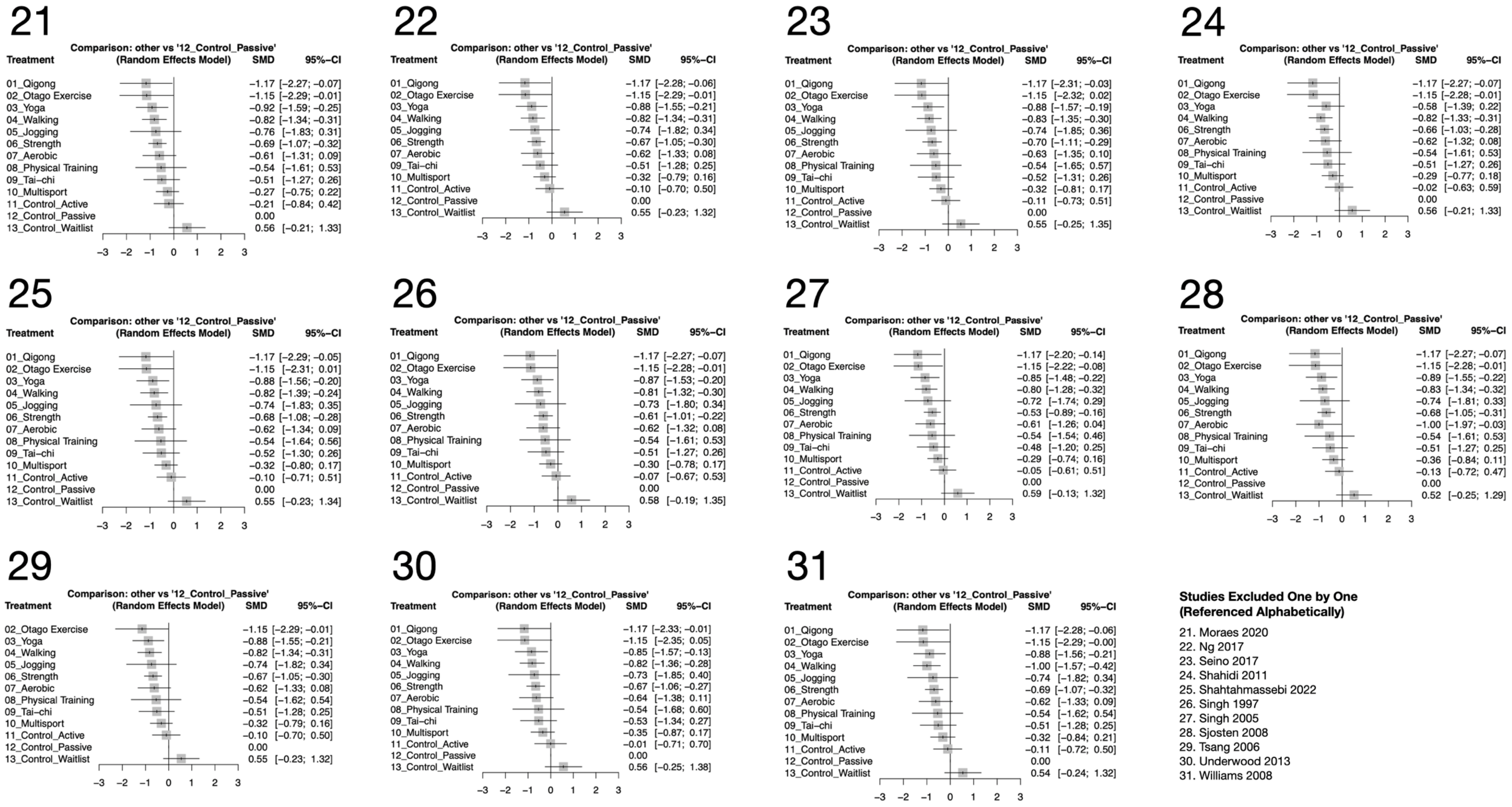

Fig. S4 -The forest plots display the results of the sensitivity analysis conducted using the one-study removal method, involving 31 studies (labeled 1 to 31). The ranking and clinical significance remain unchanged, indicating that the conclusions of our study are not affected by the inclusion or exclusion of any single study.

Fig. S5 - Forest plot displaying the improvement in depressive symptoms in elderly patients

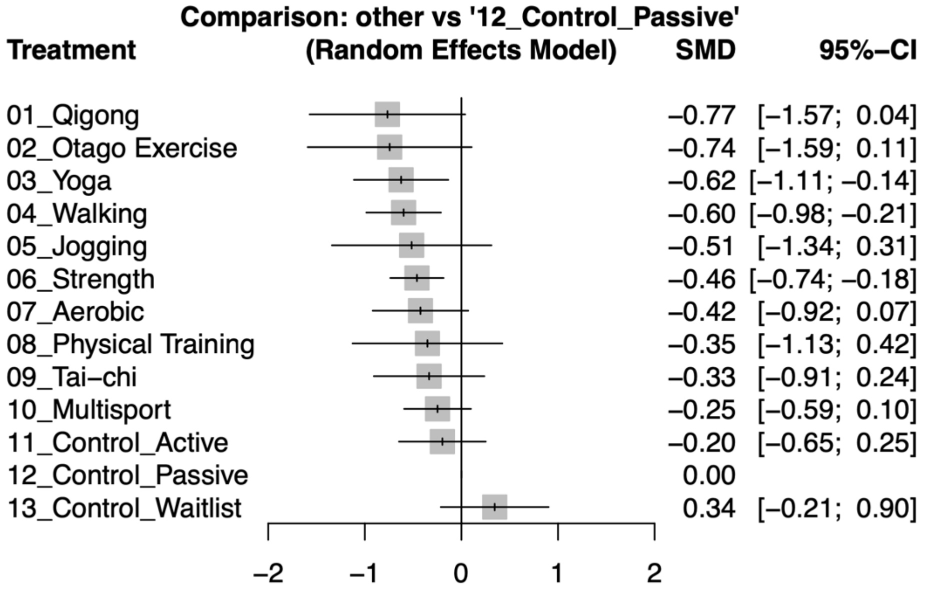

Fig. S5 - Forest plot displaying the improvement in depressive symptoms in elderly patients after receiving different types of exercise interventions, presented as standardized mean differences (SMDs). The pre-post correlation coefficient used in the calculation of data was changed from 0.8 used in Figure 3 to 0.5 in this figure as a sensitivity analysis. The ranking and clinical interpretations remained unchanged compared to Figure 3. This suggests that the conclusions of our study remain consistent despite different assumptions regarding the coefficient used for transformation.

Fig. S6 Publication Bias

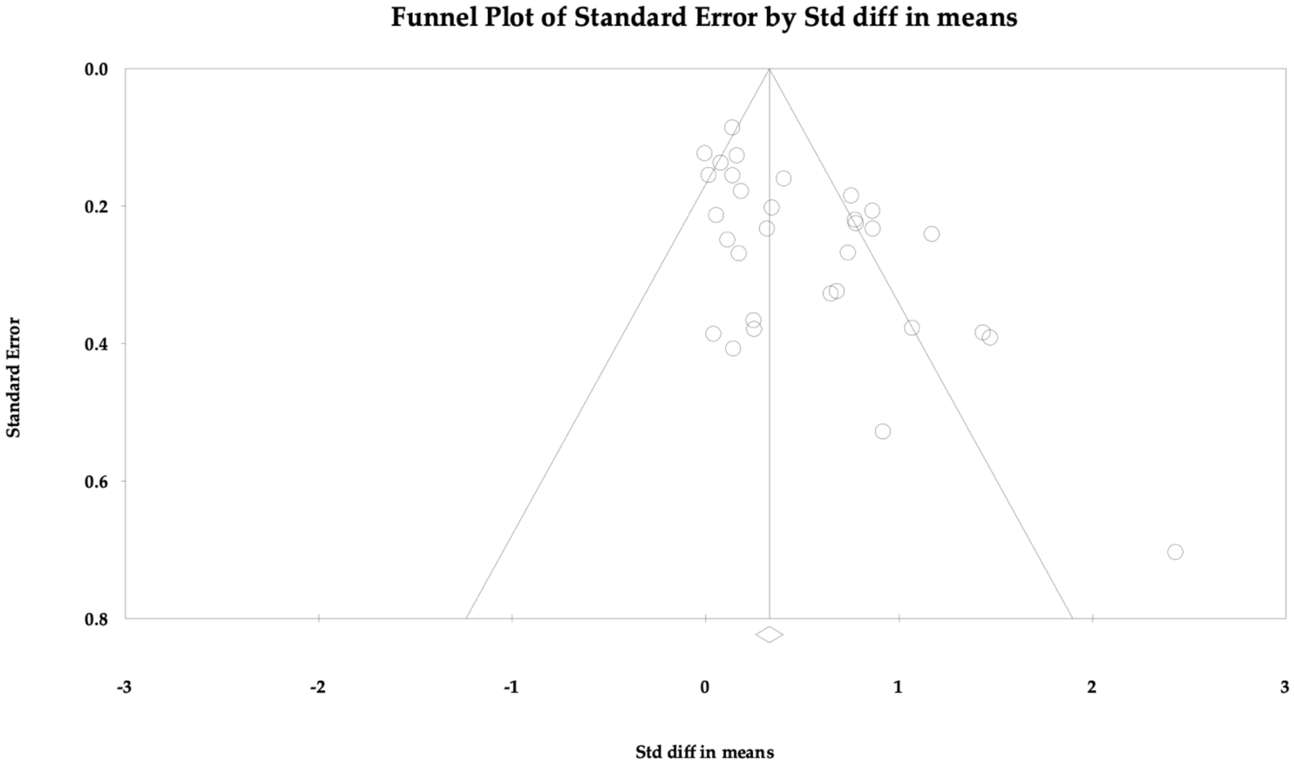

Based on the Egger's test results, the figure shows the following information: the intercept (B0) is 2.595, with a 95% confidence interval of (1.350, 3.841); the t-value is 4.263 with 29 degrees of freedom; the 1-tailed p-value (recommended) is 0.001, and the 2-tailed p-value is 0.001. These data indicate that the intercept value significantly deviates from zero, suggesting the presence of publication bias. Specifically, the very small p-value for the intercept shows statistical significance, further supporting the conclusion of bias.
